# Stereochemical Analysis of Trifluoroacetamide Derivatives Based on Through-*Space*^1^H**–**^19^F Spin–Spin Couplings

**DOI:** 10.1021/acs.joc.3c00311

**Published:** 2023-05-18

**Authors:** Yan Li, Chinatsu Ohtake, Mayuno Hotta, Hidetsugu Tabata, Kiriko Hirano, Motoo Iida, Kayo Nakamura, Kosho Makino, Tetsuta Oshitari, Hideaki Natsugari, Takenori Kusumi, Hideyo Takahashi

**Affiliations:** † Faculty of Pharmaceutical Sciences, Tokyo University of Science, 2641 Yamazaki, Noda-shi, Chiba 278-8510, Japan; ‡ Faculty of Pharma Sciences, Teikyo University, 2-11-1 Kaga, Itabashi-ku, Tokyo 173-8605, Japan; § Bruker Japan K.K., 3-9 Moriya, Kanagawa-ku, Yokohama, Kanagawa 221-0022, Japan; ∥ Research Institute of Pharmaceutical Sciences, Musashino University, 1-1-20 Shin-machi, Nishitokyo-shi, Tokyo 202-8585, Japan; ⊥ Graduate School of Pharmaceutical Science, The University of Tokyo, 7-3-1 Hongo, Bunkyo-ku, Tokyo 113-0033, Japan; # Department of Chemistry, Tokyo Institute of Technology, Meguro-ku, Tokyo 152-8551, Japan

## Abstract

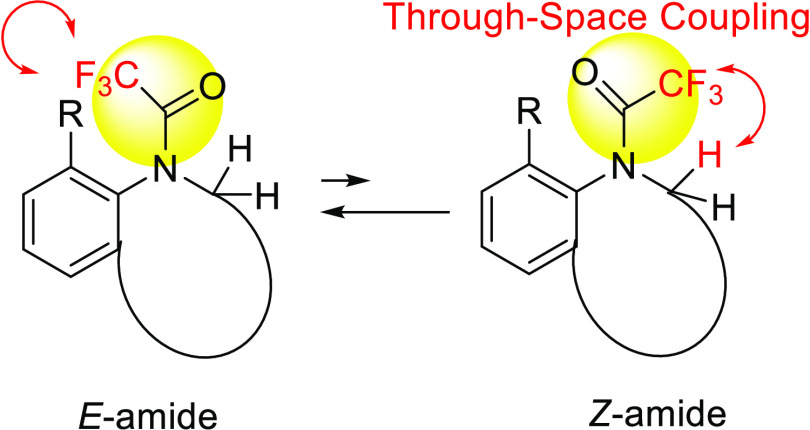

In this study, the conformational properties of tertiary trifluoroacetamides in dibenzoazepine (**1a** and **1b**) and benzodiazepine (**2a** and **2b**) derivatives, which exist as equilibrated *E*- and *Z-*amide conformers in solution, were investigated by ^1^H and ^19^F NMR spectroscopy. In all cases, one of the methylene protons neighboring the nitrogen atom of the minor conformer showed a finely split pattern due to coupling with the trifluoromethyl fluorine atoms, as confirmed by ^19^F-decoupling experiments. One-dimensional (1D) and two-dimensional (2D) ^1^H–^19^F heteronuclear Overhauser spectroscopy (HOESY) experiments were performed to confirm whether these couplings are attributable to through-*bond* spin–spin couplings (TBCs) or through-*space* spin–spin couplings (TSCs). HOESY cross-peaks between CF_3_ (^19^F) and one of the CH_2_–N protons of the minor conformers indicate that the two nuclei are spatially close to each other, thus establishing the stereochemistry of the major (*E-*) and minor (*Z-*) conformers. The *E*-amide preferences of the trifluoroacetamides are consistent with the results of density functional theory calculations and X-ray crystallographic analyses. Furthermore, the otherwise incomprehensible ^1^H NMR spectra were accurately assigned using the HOESY-determined TSCs. The ^1^H NMR assignments of the *E*- and *Z*-methyl signals of *N*,*N*-dimethyl trifluoroacetamide, the simplest tertiary trifluoroacetamide, were revised for the first time in half a century.

## Introduction

**Figure 1:**
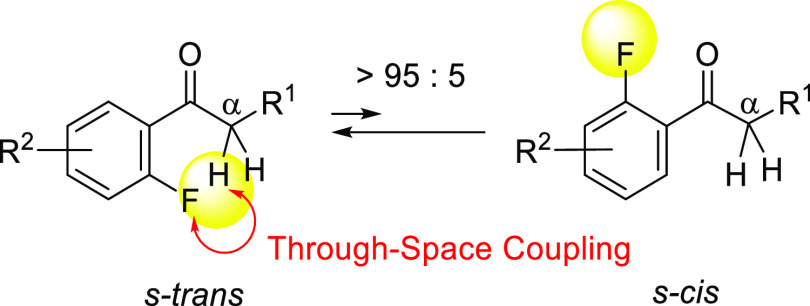
TSCs observed in 2′-fluoro-substituted acetophenone derivatives.

Fluorine, which exhibits various remarkable chemical, physical, and biological properties, plays a pivotal role in the conformation, physicochemical property modulation, and metabolic stability enhancement of a molecule.^1^ Therefore, more than 20% of known drugs contain fluorine, and this number is expected to continue to increase.^2^ Elucidating the steric and electronic effects of fluorine substituents in the molecular structures of drugs is necessary for future drug design. One of the nuclear magnetic resonance (NMR) characteristics of fluorine is through-*space* spin–spin coupling (TSC),^3^ which is observed between two atoms when either has lone-pair electrons and both are constrained at a distance smaller than the sum of their van der Waals radii. Two nuclei, such as ^19^F/^19^F, ^19^F/^1^H, and ^19^F/^13^C, can exchange spin information when in van der Waals contact through space, regardless of the number of chemical bonds separating them.^4^ In the course of our studies on the syntheses of bioactive compounds,^5^ we accidentally observed TSCs with significant magnitudes (^5^*J*_HF_ = 3.2–5.0 Hz) in the ^1^H NMR spectra of 2′-fluoro-substituted acetophenone derivatives. These TSCs revealed that 2′-fluoro-substituted acetophenone derivatives in solution exclusively form *s*-*trans* conformers (Figure 1).^5^ Additionally, the principles of TSC have been used to distinguish between amide conformations in recent years.^6^

**Figure 2:**
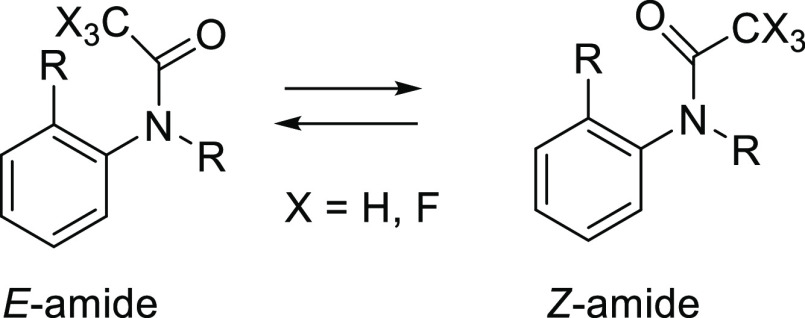
*E-* and *Z*-Conformations of tertiary acetamides.

These studies prompted us to elucidate the conformational properties of trifluoroacetamides based on their TSCs. *N*-Alkyl-*N*-aryl tertiary acetamides (X = H) are known to exist preferentially in their *E*-forms.^7^ However, little attention has been given to the conformations of trifluoroacetamides (X = F) (Figure 2).

**Figure 3:**
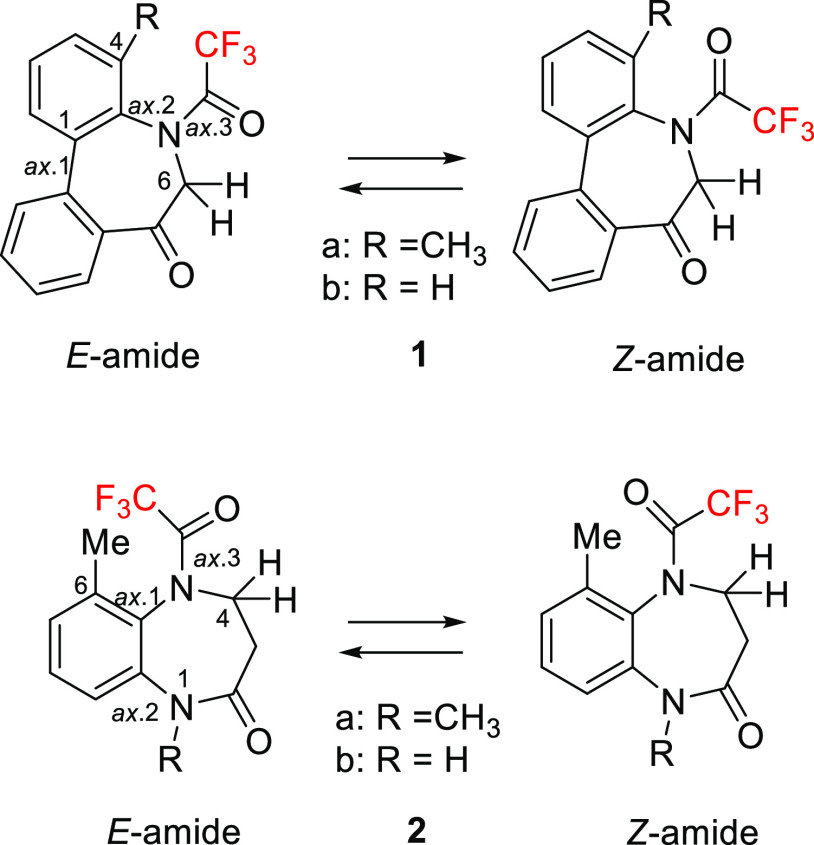
*E*- and *Z-*Conformations of compounds **1** and **2**.

Because the trifluoroacetyl group cannot be observed by ^1^H NMR spectroscopy, identifying *E*/*Z*-conformational isomers of a trifluoroacetamide in solution is difficult. Our previous study elucidated the conformational properties of atropisomeric compounds **1** and **2** containing trifluoroacetamide moieties,^8^ which are promising scaffolds for drugs. The *N*-acyl 5*H*-dibenzo[*b*,*d*] azepin-7(6*H*)-ones (**1**) were reported to exhibit immunosuppressive effects by inhibiting the potassium channels (Kv1.3, IK-1) of T cells^9^ and their conformational properties are interesting. In a similar manner, the conformational properties of 1,5-benzoxiazepin-2-ones (**2)** analogues were examined for future drug design in our previous study (Figure 3).^8b^

In this study, we report the TSCs observed in the ^1^H NMR spectra of compounds **1** and **2**, which proved to be useful for elucidating the conformations of trifluoroacetamides. Although the magnitudes of the TSCs (^5^*J*_HF_) in these compounds are smaller than those of 2′-fluoro-substituted acetophenone derivatives, we successfully distinguished TSCs from through-*bond* couplings (TBCs) using ^1^H–^19^F heteronuclear Overhauser spectroscopy (HOESY) experiments.^10^ We further used density functional theory (DFT) calculations and X-ray structural analyses to support the conformations deduced based on the observed TSCs. Furthermore, we report that TSCs provide valuable information regarding the assignment of the ^1^H NMR signals of *N*,*N*-dimethyl trifluoroacetamide (DMTFA), the spectrum of which was incomprehensible by other means.

## Results and Discussion

### Conformational Properties of Dibenzoazepine and Benzodiazepine Derivatives

**Figure 4:**
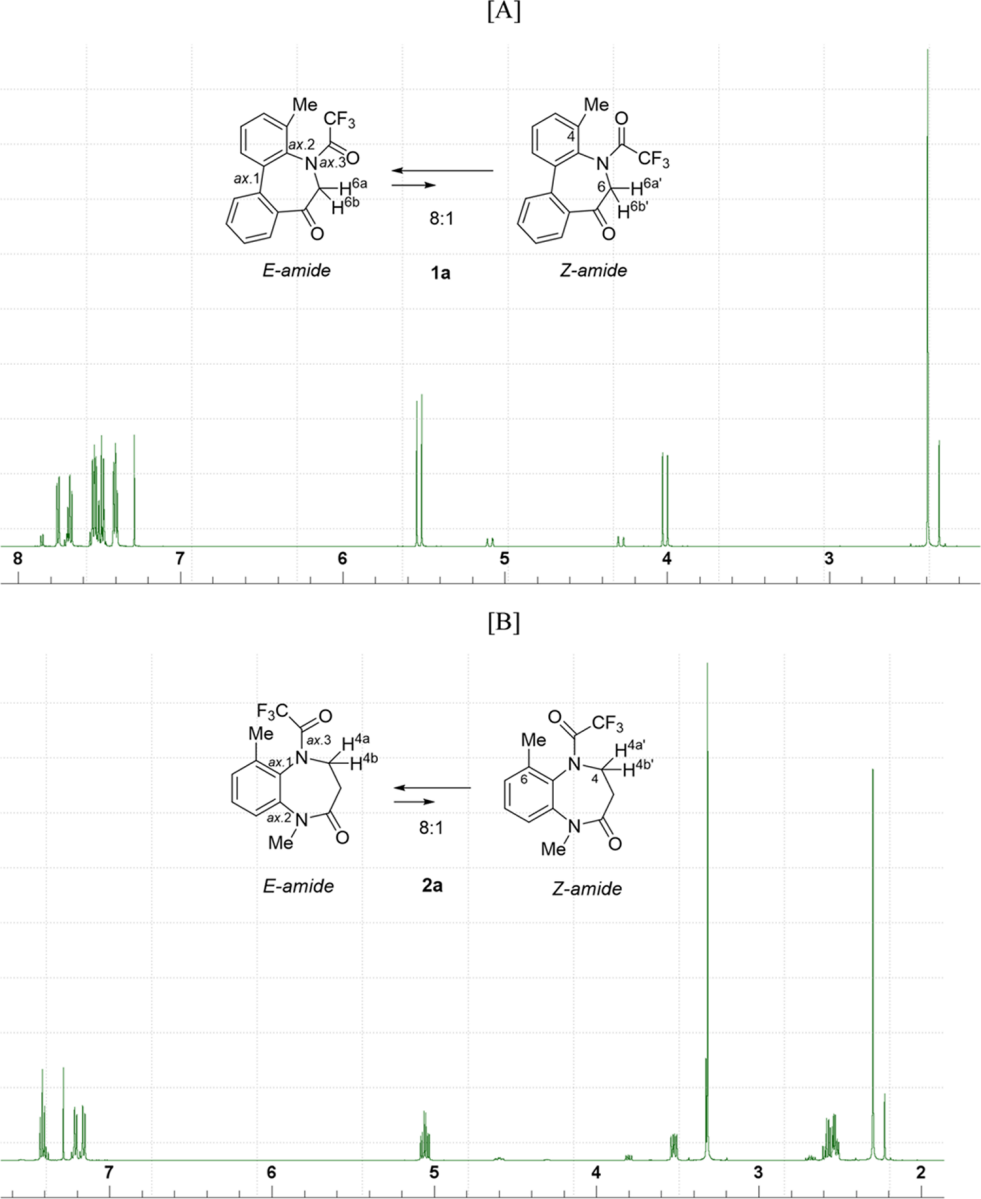
^1^H NMR spectra (400 MHz, CDCl_3_) of compounds [A] **1a** and [B] **2a**.

The ^1^H NMR spectra of **1a** and **2a** reveal that these compounds exist as equilibrium mixtures in solution (Figure 4A,B). Our previous studies revealed that their two axes (axis 1 and axis 2) move in a concerted fashion.^8^ Therefore, the seven-membered ring exists only as a pair of enantiomers (a^1^*R*, a^2^*R*) and (a^1^*S*, a^2^*S*).^8a^ Because it is unlikely that the exocyclic axis (axis 3) moves in concert with two endocyclic axes (axes 1 and 2), compound **1a** exists as *E*/*Z*-amide conformers.

In each spectrum, one of the two diastereotopic protons on C-6/C-4 in the major amide diastereomer resonates downfield from the other. This downfield shift could be due to the proximity of the amide carbonyl bond to the equatorial proton on the adjacent carbon (**1a**: 2.4 × 10^–10^ m; **2a**: 2.3 × 10^–10^ m, determined by DFT calculations).^11^ Based on this anisotropic effect of the carbonyl group, **1a** and **2a** were presumed to preferentially exist as *E*-amides; however, this presumption should be verified using spectral properties rather than anisotropy. Therefore, we investigated their spectroscopic behavior such as spin–spin couplings and nuclear Overhauser effects (NOE) between ^1^H and ^19^F.

**Figure 5:**
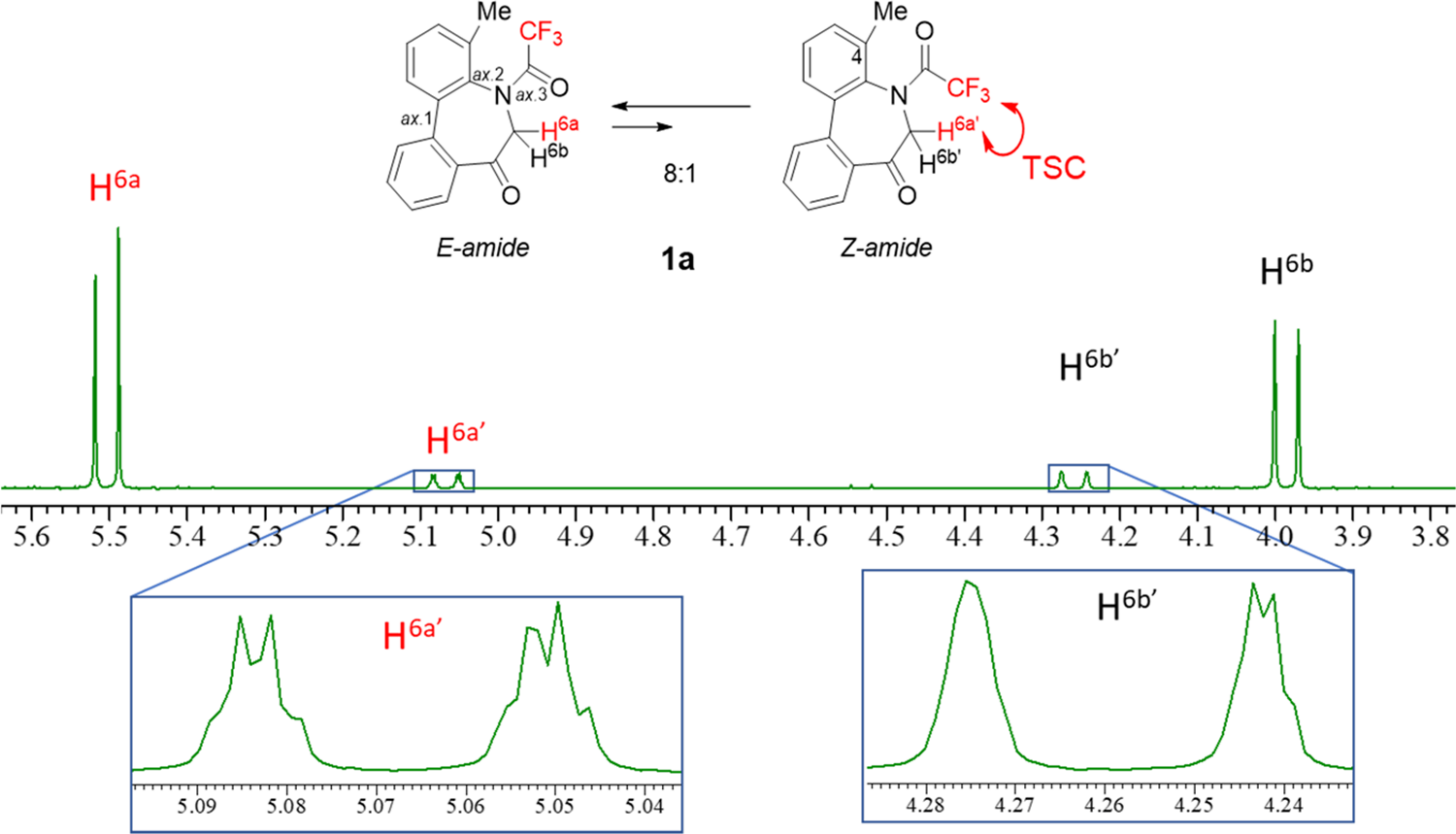
Methylene signals (above) and expanded minor peaks (below) in the ^1^H NMR spectrum (600 MHz, CDCl_3_) of **1a**.

The ^1^H NMR spectrum of compound **1a** with a methyl substituent at C4 (Figure 4A) reveals that the compound exists as an equilibrium mixture of *E*/*Z*-diastereomers in CDCl_3_ solution with an 8:1 *E*/*Z* ratio. As shown in Figure 5, the ^1^H NMR signals of the methylene protons of **1a** reveal that one of the two diastereotopic H-6 protons, H^6a^, in the major amide diastereomer resonates at 5.54 ppm, which is 1.44 ppm downfield from that of H.^7b^ As mentioned above, such a downfield shift may be ascribed to the proximity of the amide carbonyl bond to the equatorial proton (H^6a^) on the adjacent carbon (C-6),^11^ suggesting that the major diastereomer has the *E*-amide conformation. To further understand the *E*/*Z*-amide stereochemistry, coupling patterns of the methylene protons were closely investigated.

Figure 5 shows the signals of the CH_2_ protons. In contrast to the simple AB doublet (H^6a^) of the major diastereomer, H^6a^′^^ exhibits a rather complex pattern in the minor diastereomer: a doublet of quartets (dq) owing to the (1) coupling between diastereotopic methylene protons (*J*_H_^6a^′^^_H_^6b^′^^ = 19.6 Hz) and, assumingly, (2) additional coupling between CF_3_ and H^6a^′^^ (*J*_H_^6a^′^^_F_ = 1.7 Hz).

**Figure 6:**
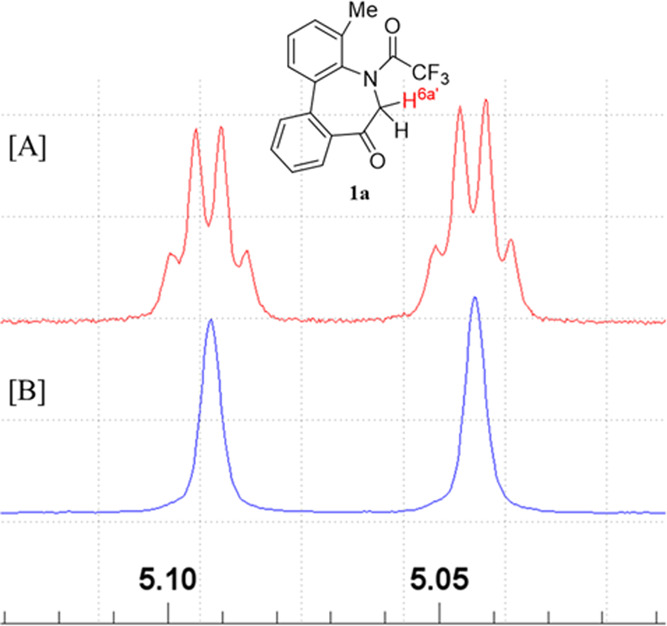
[A] Original and [B] ^1^H-{^19^F} signals corresponding to H^6a^′^^ (400 MHz, CDCl_3_) in **1a**.

**Figure 7:**
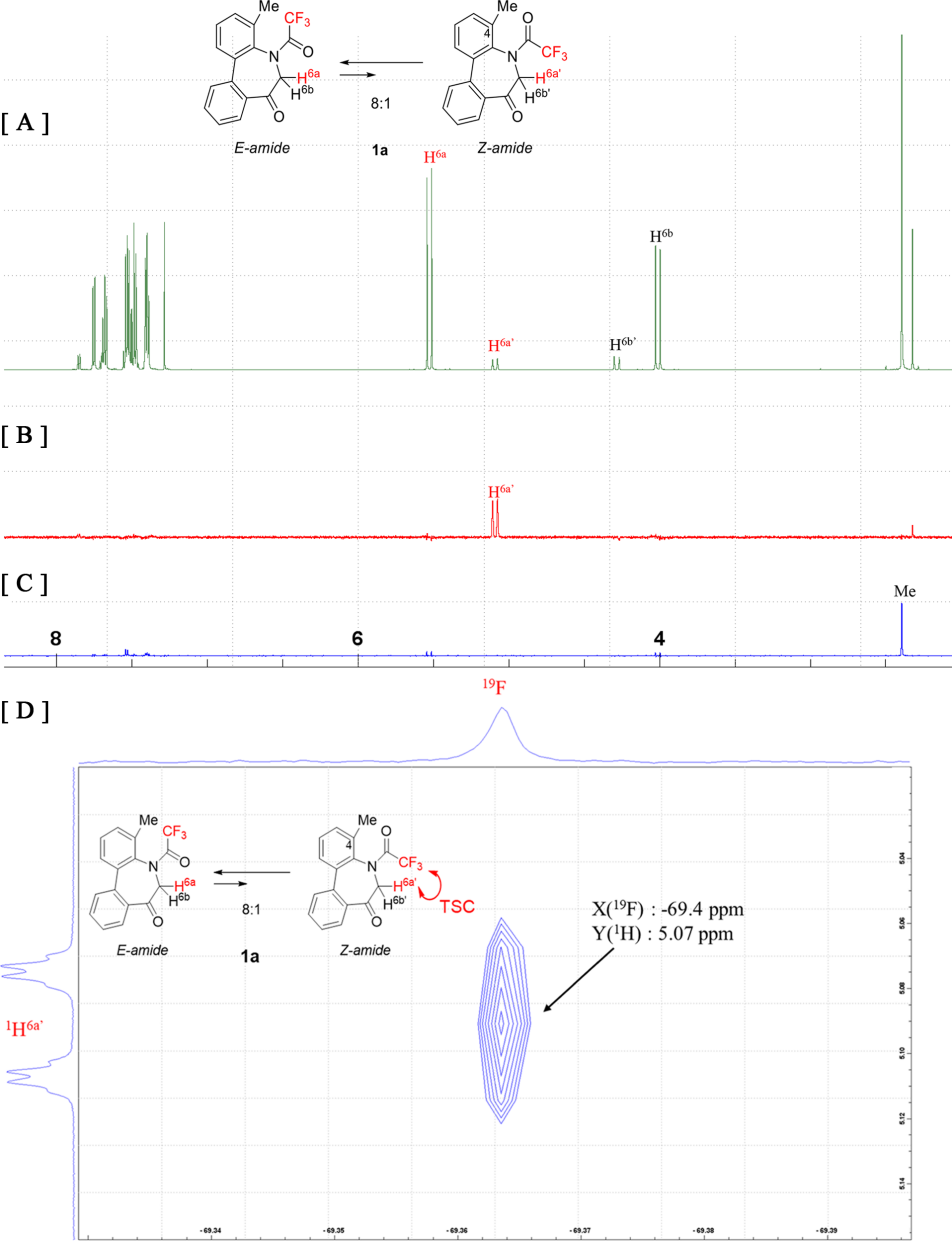

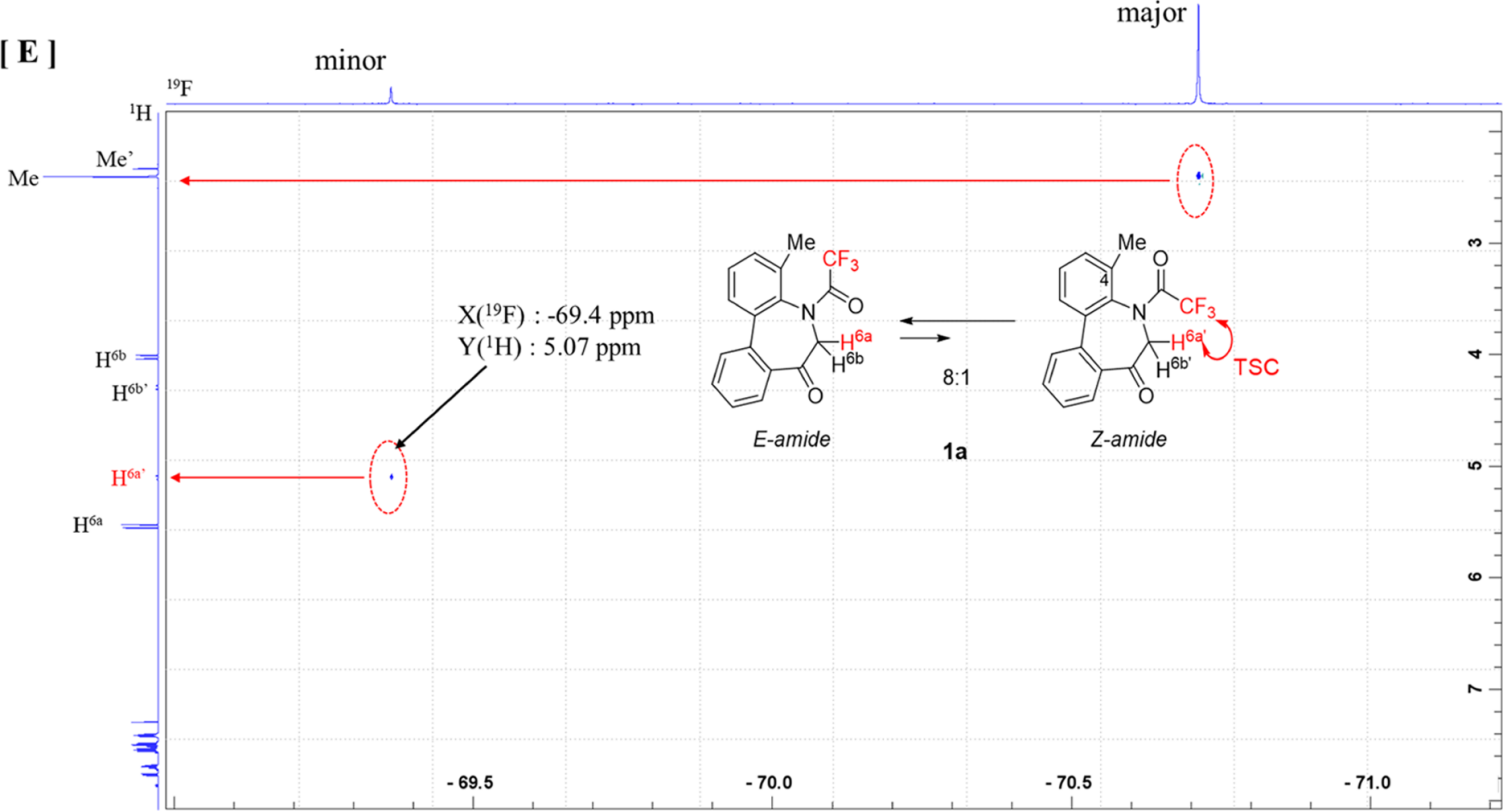
[A] ^1^H NMR spectrum (600 MHz, CDCl_3_) of **1a**. [B] 1D ^1^H–^19^F HOESY spectrum of H^6a^′^^ correlated with the minor ^19^F signal of **1a** at −69.4 ppm. [C] 1D ^1^H–^19^F HOESY spectrum of H^6a^ correlated with the major ^19^F signal of **1a** at −70.7 ppm. [D] 2D ^1^H–^19^F HOESY spectrum (600/564 MHz, CDCl_3_) showing the correlation between the minor ^19^F signal (−69.4 ppm) and the minor ^1^H signal (H^6a^′^^). [E] 2D ^1^H–^19^F HOESY, full spectrum (600/564 MHz, CDCl_3_).

A ^19^F-decoupling experiment was conducted to confirm the origin of small couplings involving H^6a^′^^. As shown in Figure 6B, irradiation of ^19^F simplified the signal patterns. However, concluding whether such small couplings are due to TSC or TBC is difficult. Therefore, one-dimensional (1D) and two-dimensional (2D) ^1^H–^19^F heteronuclear NOE spectroscopy (HOESY) experiments were conducted on **1a** (Figure 7) to differentiate between TSC and TBC. These spectra exhibit NOE correlation signals between ^19^F (−69.4 ppm: minor ^19^F signal) and ^1^H (5.07 ppm) for the minor conformer, indicating that the corresponding two atoms (H^6a^′^^ and F) in the minor amide diastereomer are in sufficiently close proximity to cause spatial spin–spin interactions. These experiments provided strong evidence that TSC is responsible for the splitting observed for H^6a^′^^ (*J*_H_^6a^′^^_F_ = 1.7 Hz). Additionally, a correlation between the CH_3_ group at the C4 position of the phenyl ring and the F of CF_3_ can be observed in the major conformer (Figure 7C,E). Unfortunately, the broad signal of the CH_3_ group at the C4 position in the 1D ^1^H NMR spectrum is not a suitable indicator of TSC. However, the carbon signal of the CH_3_ group at the C4 position is split into a quartet due to the TSC with CF_3_ (Supporting Information, Page S11).

**Figure 8:**
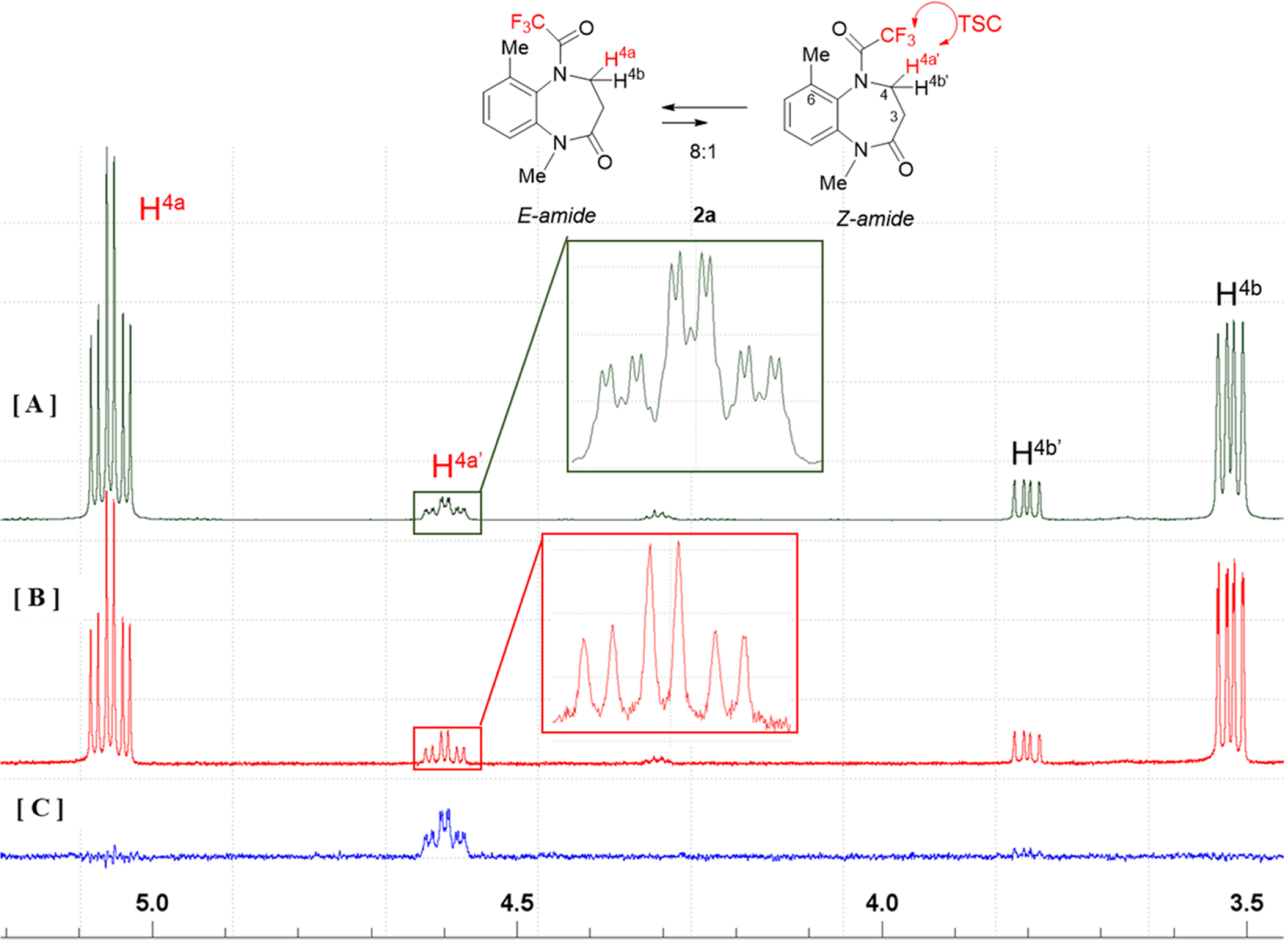
600 MHz ^1^H NMR spectrum of **2a** (CDCl_3_): [A] H^4a^/H^4b^ and H^4a^′^^/H^4b^′^^ signals, [B] ^1^H-{^19^F} spectrum, and [C] 1D ^1^H–^19^F HOESY (irradiated at −69.2 ppm: minor ^19^F signal) spectrum.

Compound **2a**(8b) with a methyl substituent at C6, which has similar axial chirality to compound **1a**, was next examined. As shown in Figure 8A, the signal corresponding to H^4a^′^^ in the minor amide diastereomer was observed as a triplet of doublet of quartets (tdq) due to coupling to diastereotopic methylene protons (H^4b^′^^, H^3^, H^3^′^^) and the additional coupling to CF_3_. As was observed for compound **1a**, ^1^H-{^19^F} ^1^H NMR and ^1^H–^19^F HOESY experiments were conducted for compound **2a**. As shown in Figure 8B, a simplified H^4a^′^^ signal was observed when the ^19^F signal was irradiated, confirming that the CF_3_ (*J*_H_^4a^′^^_F_ = 1.6 Hz) is responsible for the observed coupling. Figure 8C shows a 1D ^19^F HOESY (o2p: −69.2 ppm: the minor ^19^F resonance) spectrum of compound **2a**, which reveals that H^4a^′^^ and F in the minor amide diastereomer are correlated. These experiments reveal that the two atoms (H^4a^′^^ and F) of the minor amide diastereomer are spatially close and that compound **2a** exists as the *E*-amide^12^ rather than the *Z*-amide in solution. Similar to that of compound **1a**, in the HOESY spectrum of **2a**, a correlation between the CH_3_ group at the C6 position and CF_3_ is observed for the major conformer (Supporting Information, Page S9), although a clear splitting pattern was not observed due to signal broadening. However, the C6 carbon signal is split into a quartet due to the TSC with CF_3_ (Supporting Information, Page S12).

**Figure 9:**
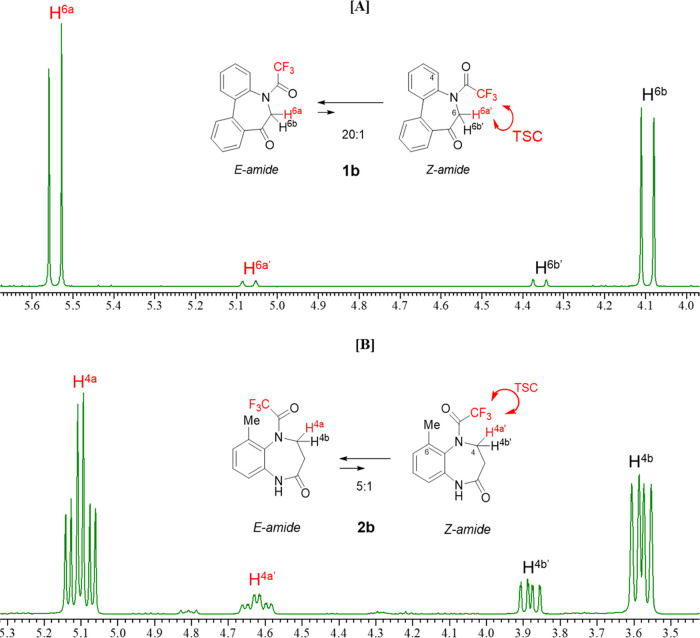
Signals corresponding to the methylene protons of [A] **1b** and [B] **2b** (400 MHz, CDCl_3_).

**Figure 10:**
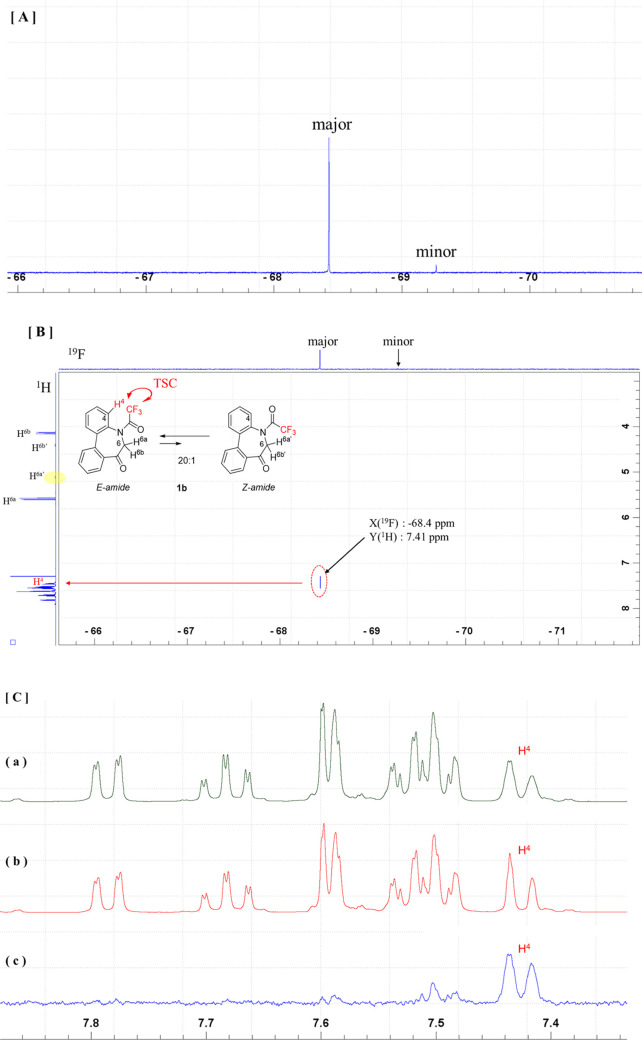
[A] ^19^F NMR spectrum of **1b** (564 MHz, CDCl_3_). [B] 2D ^1^H–^19^F HOESY NMR spectrum (600/564 MHz, CDCl_3_) of compound **1b**. [C] 400 MHz ^1^H NMR spectrum of **1b** (CDCl_3_): (a) Original ^1^H NMR protons signals of **1b** and (b) its ^1^H-{^19^F} spectrum and (c) 1D ^19^F HOESY (Irradiated at −68.4 ppm: major ^19^F signal) spectrum of **1b**.

We next investigated the spectral properties of **1b** and **2b**. The ^1^H NMR spectra of these compounds show that they exist as equilibrium mixtures of *E*- and *Z*-amides in solution (**1b**: 20:1, **2b**: 5:1) (Figure 9A,B). In each spectrum, one (H^6a^ of **1b**, H^4a^ of **2b**) of the two diastereotopic proton resonances in the major amide diastereomer is located downfield from its partner (H^6b^ of **1b**, H^4b^ of **2b**). As mentioned above, such a downfield shift was deduced to be due to the proximity of the amide carbonyl bond to the equatorial proton on the adjacent carbon (C^6^/C^4^).^11^ Furthermore, based on this anisotropic effect of the carbonyl group, **1b** and **2b** were supposed to preferentially exist as *E*-amides. Fine ^1^H–^19^F couplings were observed in the methylene signals of the minor amide diastereomers. To confirm that these couplings are due to TSC, the 1D and 2D ^1^H–^19^F HOESY spectra of these compounds were analyzed (Figure 10).

As shown in Figures 9A and 10A, **1b** exhibits a significantly small (∼1/20) minor conformer ratio such that no distinct correlation between CF_3_ and H^6a^′^^ was observed in the 1D or 2D ^1^H–^19^F HOESY spectrum. However, the 2D spectrum exhibits an apparent cross-peak between the major ^19^F signal and the aromatic proton signal (7.41 ppm) (Figure 10B). This doublet signal is rather broad (Figure 10C(a)) but sharpens through ^19^F decoupling (Figure 10C(b)), which indicates that CF_3_ is coupled with the spatially close aromatic proton (H^4^) through TSC. This notion was further verified through 1D HOESY spectroscopy, where the corresponding spectrum (Figure 10C(c)) exhibits a correlation between the major CF_3_ signal and the aromatic proton (H^4^).

**Figure 11:**
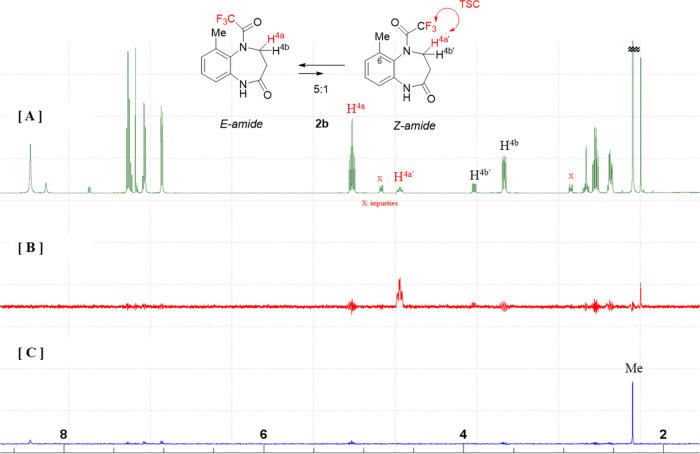
[A] ^1^H NMR spectrum, [B] 1D HOESY spectrum (Irradiated at: −69.1 ppm: minor ^19^F signal), and [C] 1D HOESY spectrum (Irradiated at: −70.6 ppm: major ^19^F resonance) of **2b** (400 MHz, CDCl_3_).

In contrast to **1b**, the 1D HOESY spectrum of **2b** exhibits distinct correlations between the minor ^19^F resonance and the H^4a^′^^ resonance of the minor conformer (Figure 11B) and between the major ^19^F resonance and the aromatic Me resonance of the major conformer (Figure 11C), which implies that the major and minor conformers have *E*-amide and *Z*-amide structures, respectively, and, at the same time, the H^4a^′^^ peak broadening is due to TSC caused to the adjacent CF_3_. Similar to that of compound **2a**, in the HOESY spectrum of **2b**, the correlation between the CH_3_ group at the C6 position and CF_3_ is observed for the major conformer (Figure 11C, Supporting Information, Page S9), and a clear splitting pattern was not observed due to signal broadening. However, the CH_3_ carbon at C6 signal is split into a quartet due to the TSC with CF_3_ (Supporting Information, Page S14).

It is worth mentioning that the coupling constants (^5^*J*_HF =_ 1.4–1.8 Hz) (Table 1) associated with through-*space* coupling in compounds **1** and **2** are smaller than those in 2′-fluoro-substituted acetophenones (^5^*J*_HF =_ 3.2–5.0 Hz), as reported in our previous study (Figure 1).^5^ The TBC constants (^5^*J*_HF_) of most compounds are generally less than 1 Hz, which is similar in magnitude to those observed for compounds **1** and **2**. However, it should be noted that, although ^1^H–^19^F coupling was observed in one of the diastereotopic protons (H^6a^′^^ and H^4a^′^^ in compounds **1** and **2**, respectively), no such couplings were observed for other diastereotopic protons (H^6b^ in compound **1** and H^4b^ in compound **2**), which are both five bonds apart from the F in CF_3_ group. Therefore, we conclude that these compounds have negligible TBC constants, ^5^*J*_HF_.

**Table 1:**
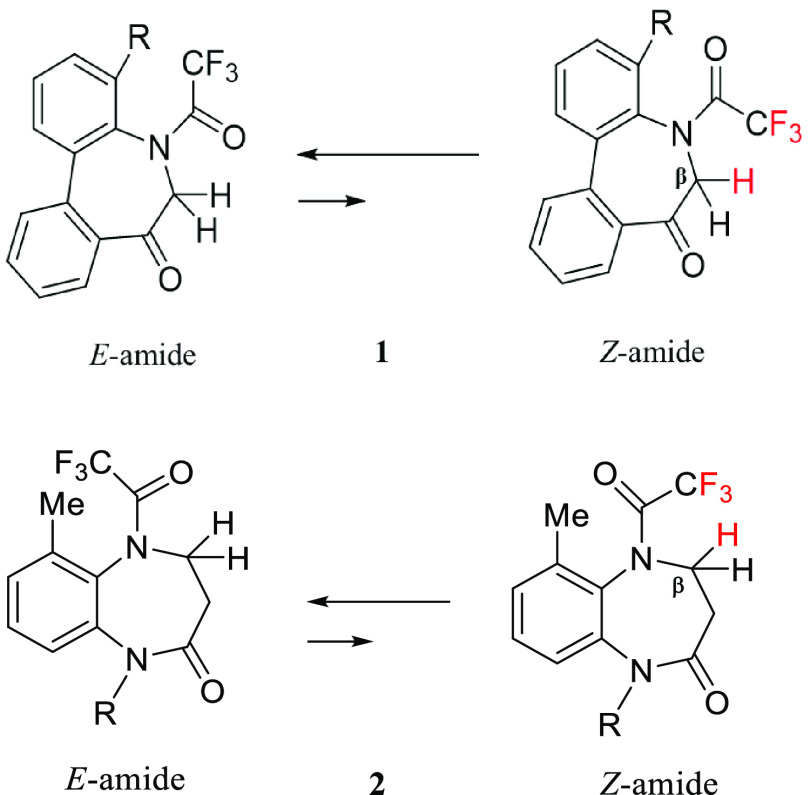
TSC Constants and *E*/*Z* Ratios of Compounds **1** and **2** Calculated Using Density Functional Theory at the B3LYP/6-31G(d)/SM8:CHCl_3_ Level

compound	R	*J* (H^β^, F) (Hz)	*J* (C^β^, F) (Hz)	*E*:*Z*
**1a**	–CH_3_	1.7	2.9	2:1
**1b**	–H	1.8	3.9	9:1
**2a**	–CH_3_	1.6	3.9	4:1
**2b**	–H	1.4	4.3	2:1

As mentioned above, we clarified the preference for the *E*-amide diastereomers of compounds **1** and **2**. To further understand the stability of *E*-amide compared to *Z*-amide, compounds **1** and **2** were analyzed using DFT calculations. These compounds were subjected to conformer search using MMFF^11^ followed by structural optimization at the B3LYP/6-31G(d) level^13^ with solvent (CHCl_3_) included using the SM8 model. Conformer distributions were calculated based on free energies at 297.5 K obtained by frequency analysis at the same level of theory.

As expected, DFT calculations suggest that the *E*-amide diastereomers of **1a**, **1b**, **2a**, and **2b** are more stable than the corresponding *Z-*amide diastereomers. Furthermore, using the calculated energy differences (Δ*G*) between the *E* and *Z* conformers, the *E*/*Z* ratios based on the Boltzmann distribution were also calculated (Table 1), which revealed that the compounds exist predominantly as *E* diastereomers.

The calculated H^β^···F and C^β^···F distances in the *Z*-amide diastereomers of compounds **1a**, **1b**, **2a**, and **2b** are shown in Table 2. In all cases, the H^β^–F and C^β^–F internuclear distances are smaller than the sums of the fluorine and hydrogen (∼2.7 × 10^–10^ m) and fluorine and carbon (∼3.2 × 10^–10^ m)^14^ van der Waals radii, respectively. Similarly, the calculated H of CH_3_^4/6^···F and C of CH_3_^4/6^···F internuclear distances in the *E-*amide diastereomers in the *E-*amide diastereomers of compounds **1a**, **b** and **2a**, **b** are shown in Table 3. The calculated internuclear distances from CF_3_ to CH_3_ are smaller than the sum of the van der Waals radii (Table 3).

**Table 2:** H^β^···F and C^β^···F Internuclear Distances in the *Z-*Amide Diastereomers of Compounds **1a**, **b** and **2a**, **b** Calculated at the B3LYP/6-31G(d)/SM8:CHCl_3_ Level

		internuclear distance (10^–10^ m)	
compound	R	H^β^···F	C^β^···F
**1a**	–CH_3_	2.5	2.9
**1b**	–H	2.5	2.9
**2a**	–CH_3_	2.3	3.1
**2b**	–H	2.3	3.1

**Table 3:** H of CH_3_^4/6^···F and C of CH_3_^4/6^···F Internuclear Distances in the *E-*Amide Diastereomers of Compounds **1a**, **b** and **2a**, **b** Calculated at the B3LYP/6-31G(d)/SM8:CHCl_3_ Level

		internuclear distance (10^–10^ m)	
compound	R	H···F	C···F
**1a**	–CH_3_	2.4	3.1
**1b**	–H	2.6	3.0
**2a**	–CH_3_	2.4	3.2
**2b**	–H	2.5	3.1

**Figure 12:**
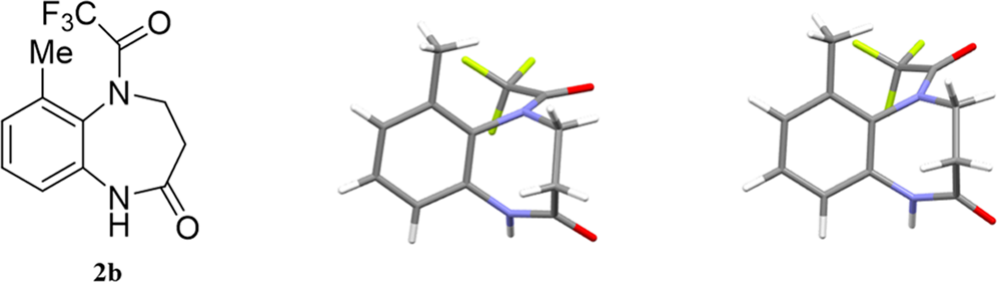
(Left) X-ray crystal structure of **2b**. (Right) Optimized structure of **2b** calculated at the B3LYP/6-31G(d), SM8:CHCl_3_ level of theory (right).

Compound **2b** was examined by X-ray crystallography because it formed single crystals, which revealed that only the *E-*amide diastereomer is present in the crystals (Figure 12, left). Figure 12 (right) shows the DFT-optimized *E-*amide diastereomer of **2b** for comparison, which was calculated by including the effect of CDCl_3_. The calculated structure is very similar to that of the solid state.

The reason for the preference of the *E*-amide conformation of a trifluoroacetamide remains poorly understood. We speculate that electronic and steric repulsions between the π system of the arene and the lone pairs on the carbonyl oxygen of the amide might destabilize the *Z*-form.^7c^

### Assignment of *N*,*N*-Dimethyl Trifluoroacetamide (DMTFA)

**Figure 13:**
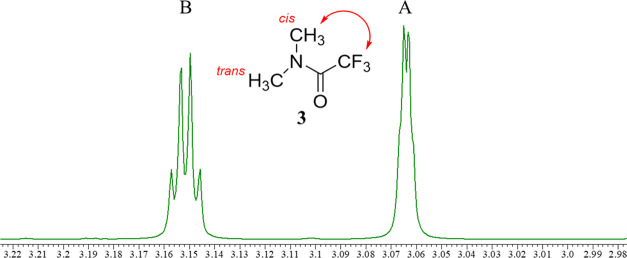
^1^H NMR spectrum of **3** (400 MHz, CDCl_3_).

As discussed above, TSCs provide valuable information not only on the stereochemistry of compounds but also for assigning the ^1^H NMR signals of trifluoroacetamides. To prove this supposition, we examined the ^1^H NMR signals of DMTFA (**3**), the simplest tertiary trifluoroacetamide; its spectrum consists of two methyl signals (*cis* to CF_3_ and *trans* to CF_3_) (Figure 13).

**Figure 14:**
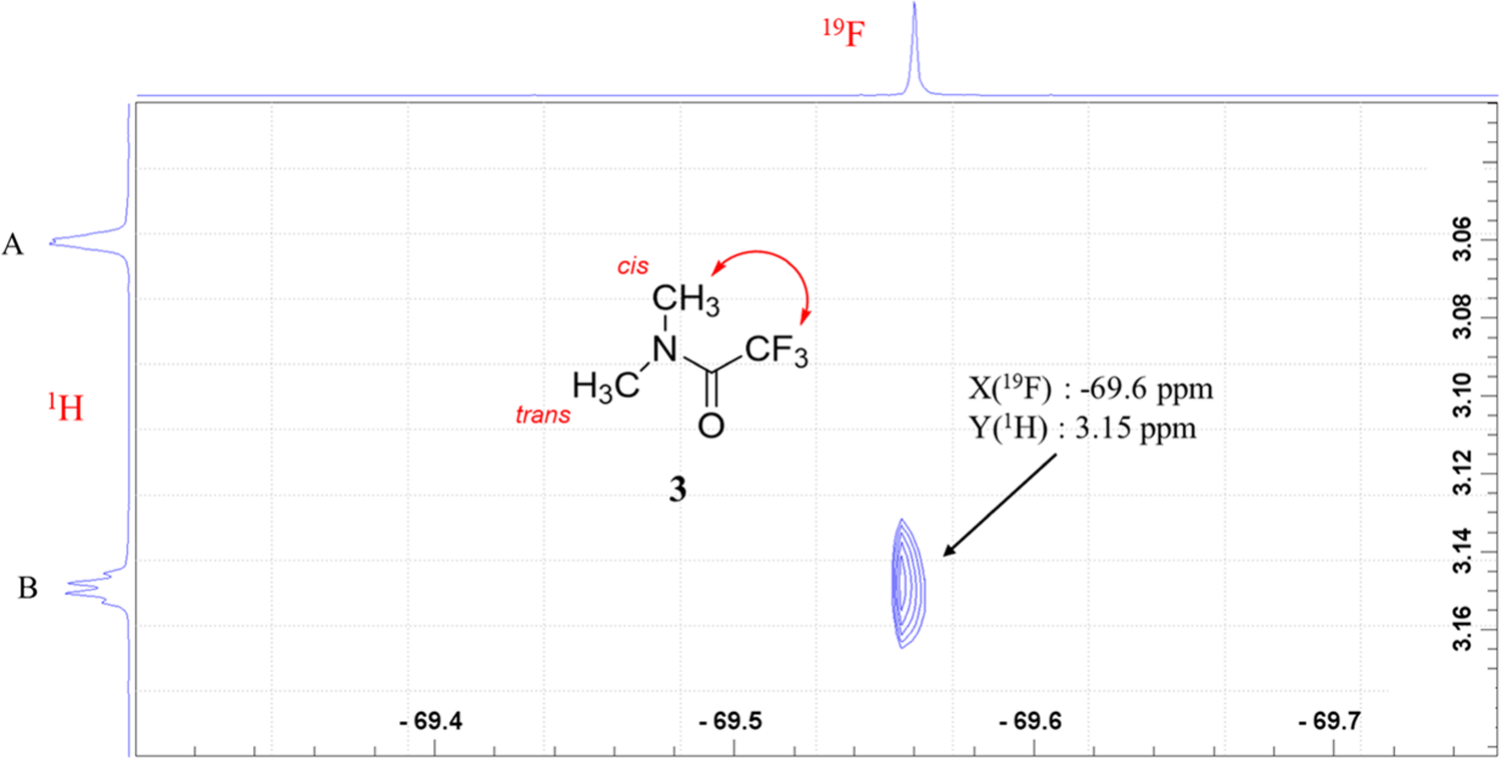
2D ^1^H–^19^F HOESY NMR spectrum (600/564 MHz, CDCl_3_) of **3**.

Signal B (3.15 ppm), at a lower chemical shift than signal A (3.06 ppm), is conventionally assigned to the *trans-*methyl group by anticipating the anisotropic effect of the amide carbonyl group. However, peak B is as a quadruplet assumed to arise through TSC from CF_3_. Thus, peak B should be assigned to the *cis*-methyl group, which is inconsistent with the results of a previous study.^14^ To prove that the coupling is not solvent-dependent, we replaced the solvent with CD_3_OD and found that similar coupling was observed (Supporting Information, Page S32). Therefore, a 2D ^1^H–^19^F HOESY experiment was performed on **3**. As shown in Figure 14, the NOE cross-peak was observed between ^19^F (−69.6 ppm) and ^1^H (3.15 ppm), from which we conclude that the signal at low magnetic field (peak B) must correspond to the *cis*-methyl group, with peak A corresponding to the *trans*-methyl group.

Assignments based on downfield shifts caused by the anisotropic effects of carbon groups are usually reliable for ordinary tertiary acetamides. However, this study revealed that TSCs provide major clues when discussing the conformations of tertiary trifluoroacetamides.^15^

## Conclusions

Through-*space* couplings (TSCs) were observed in the NMR spectra of trifluoroacetamides **1** and **2**, and the preference for the *E*-amide conformation was elucidated. While the magnitudes of the coupling constants were not very large (1.4–1.8 Hz), ^1^H–^19^F HOESY experiments revealed that the H–F couplings observed in the ^1^H NMR spectra of compounds **1** and **2** are TSCs. X-ray structural analyses and DFT calculations confirmed that **1** and **2** exist in the *E*-amide in preference to the *Z*-amide, confirming the accuracy of our TSC-based stereochemical analyses. Observing TSCs provide a guide for assigning fluorinated compounds such as DMTFA. Since the trifluoroacetyl group is not directly observed by ^1^H NMR spectroscopy, and because the conformation of a bioactive molecule determines how it interacts with its active site, TSCs are expected to be useful for elucidating the conformations of medicinally important bioactive compounds in the future.

## Experimental Section

### General Methods

NMR spectra were recorded on a spectrometer at 400 or 600 MHz for ^1^H NMR, and at 100 or 150 MHz for ^13^C NMR. ^19^F NMR spectra were recorded at 564 MHz. Chemical shifts are given in parts per million (ppm) downfield from tetramethylsilane as the internal standard, and coupling constants (*J*) are reported in hertz (Hz). Splitting patterns are abbreviated as follows: singlet (s), doublet (d), triplet (t), quartet (q), triplet of doublet of quartet (tdq), doublet of quartet (dq), doublet of doublet of doublets (ddd), multiplet (m), and broad (br). Structural assignments were made with additional information obtained from the results of gCOSY, gHSQC, and gHMBC experiments. IR spectra were recorded on a Fourier transform infrared (FT-IR) spectrometer equipped with ATR (diamond). High-resolution mass spectra (HRMS) were recorded on a time-of-flight mass spectrometry (TOF-MS) instrument in the ionization mode of electrospray ionization (ESI) and atmospheric-pressure chemical ionization (APCI). Analytical thin-layer chromatography was performed on precoated, glass-backed silica gel plates. Column chromatography was performed using silica gel (45–60 μm). Extracted solutions were dried over anhydrous MgSO_4_ or Na_2_SO_4_. Solvents were evaporated under reduced pressure.

#### 1,6-Dimethyl-1,3,4-trihydro-2*H*-*N*-trifluoroacetyl-1,5-benzodiazepin-2-one (**2a**)

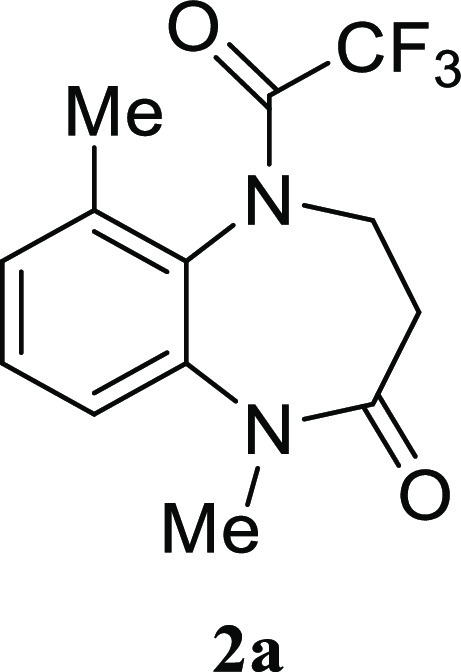



1,6-Dimethyl-1,3,4,5-tetrahydro-2*H*-1,5-benzodiazepin-2-one^7^^b^ (50 mg, 0.263 mmol) was dissolved in CH_2_Cl_2_ (3 mL) under argon, after which triethylamine (44.4 μL, 0.316 mmol) was added with stirring. Trifluoroacetic anhydride (44.5 μL, 0.316 mmol) was then added at 0 °C, followed by further stirring for 1 h. The mixture was extracted with CH_2_Cl_2_, and the extract was washed with brine, dried over Na_2_SO_4_, filtered, and concentrated in vacuo. The concentrate was purified by silica gel column chromatography (hexane/EtOAc = 1:3) to afford compound **2a** (72.9 mg, 97%).

^1^H NMR (400 MHz, CDCl_3_) *E*-isomer: δ 7.37–7.44 (m, 1H), 7.23–7.15 (m, 2H), 5.04 (td, *J* = 12.9, 6.2 Hz, 1H), 3.49–3.55 (m, 1H), 3.33–3.29 (m, 3H), 2.72–2.48 (m, 2H), 2.29 (s, 3H); *Z*-isomer: δ 7.37–7.44 (m, 1H), 7.23–7.15 (m, 2H), 4.58 (tdq, *J* = 12.8, 5.7, 1.8 Hz, 1H), 3.77–3.83 (ddd, *J* = 7.3 Hz, 1H), 3.33–3.29 (m, 3H), 2.72–2.48 (m, 2H), 2.21 (s, 3H); ^13^C{^1^H} NMR (100 MHz, CDCl_3_) *E*-isomer: δ 169.8, 156.7 (C–F, ^2^*J*_C–F_ = 35.6 Hz), 143.0, 138.2, 130.1, 129.7, 128.6, 120.5, 116.0 (C–F, ^1^*J*_C–F_ = 288.0 Hz), 48.2, 34.4, 33.2, 17.2 (C–F, ^6^*J*_C–F_ = 1.9 Hz); Z-isomer: δ 169.5, 141.5, 137.0, 130.5, 129.5, 129.0, 121.1, 48.6 (C–F, ^4^*J*_C–F_ = 3.9 Hz), 34.7, 33.5; ^19^F NMR (564 MHz, CDCl3) *E*-isomer: δ −70.4; *Z*-isomer: δ −69.2. IR-ATR: 1693, 1663 cm^–1^. HRMS (APCI, *m*/*z*) calcd for: C_13_H_13_N_2_O_2_F_3_Na [(M + Na)^+^]: 309.0822, found 309.0821.

#### 6-Methyl-1,3,4-trihydro-2*H*-*N*-trifluoroacetyl-1,5-benzodiazepin-2-one (**2b**)

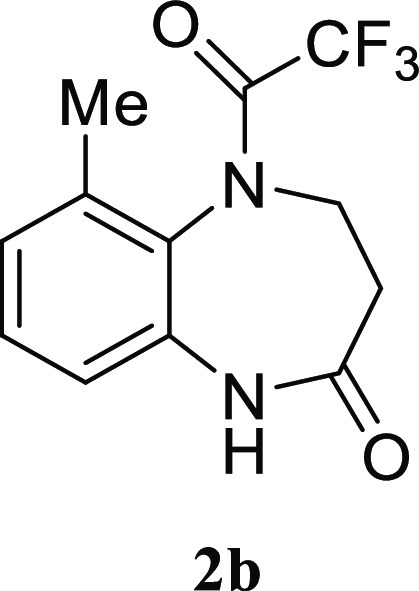



6-Methyl-1,3,4,5-tetrahydro-2*H*-1,5-benzodiazepin-2-one^7^^b^ (20 mg, 0.183 mmol) was dissolved in CH_2_Cl_2_ (3 mL) under argon, after which triethylamine (47.8 μL, 0.341 mmol) was added with stirring. Trifluoroacetic anhydride (48.0 μL, 0.341 mmol) was then added at 0 °C, and stirring was continued for 20 h. The mixture was extracted with CH_2_Cl_2_, and the extract was washed with brine, dried over Na_2_SO_4_, filtered, and concentrated in vacuo. The concentrate was purified by silica gel column chromatography (hexane/EtOAc = 1:1) to give compound **2b** (33.3 mg, 80%).

^1^H NMR (400 MHz, CDCl_3_) *E*-isomer: δ 8.42 (br, 1H), 7.29–7.37 (m, 1H), 7.15–7.21 (m, 1H), 7.01 (d, *J* = 7.8 Hz, 1H), 5.10 (td, *J* = 13.3, 6.0 Hz, 1H), 3.53–3.62 (m, 1H), 2.61–2.72 (m, 1H), 2.44–2.55 (m, 1H), 2.29 (s, 3H); *Z*-isomer: δ 8.42 (br, 1H), 7.29–7.37 (m, 1H), 7.15–7.21 (m, 1H), 7.01 (d, *J* = 7.8 Hz, 1H), 4.62 (tdq, *J* = 12.8, 6.0, 1.4 Hz, 1H), 3.85–3.92 (ddd, *J* = 8.0, 1H), 2.72–2.81 (m, 1H), 2.44–2.55 (m, 1H), 2.21 (s, 3H); ^13^C{^1^H} NMR (100 MHz, CDCl_3_) *E*-isomer: δ 172.2, 157.2 (C–F, ^2^*J*_C–F_ = 35.6 Hz), 138.4, 137.5, 130.2, 129.3, 128.4, 120.4, 115.7 (C–F, ^1^*J*_C–F_ = 288.1 Hz), 47.9, 32.8, 17.3 (C–F, ^6^*J*_C–F_ = 1.9 Hz); *Z*-isomer: δ 171.7, 135.8, 129.6, 128.6, 121.0, 48.3 (C–F, ^4^*J*_C–F_ = 3.9 Hz), 33.0, 17.4; ^19^F NMR (564 MHz, CDCl_3_) *E*-isomer: δ −70.7; *Z*-isomer: δ −69.1. IR-ATR: 3142, 1698, 1666 cm^–1^. HRMS (APCI, *m*/*z*) calcd for: C_12_H_11_N_2_O_2_F_3_Na [(M + Na)^+^]: 295.0665, found 295.0664.

## Data Availability

The data underlying this study are available in the published article and the Supporting Information.

## Supporting Information Available

H^6a^ region of the ^1^H NMR spectrum of **1**; H^4a^ of the ^1^H NMR spectrum of **2**; H^6^ and H^4^ regions of the ^1^H NMR spectra of **1b** and **2b**, respectively; ^1^H-{^19^F} spectra of **1b** and **2b**; ^19^F spectra of **1a-b** and **2a-b**; ^1^H–^19^F HOESY experiments; 2D-^1^H–^19^F HOESY experiments; X-ray crystal data for **2b**; ^1^H-, ^13^C-, and 2D-NMR spectra; DFT calculations; 1D and 2D HOESY experimental conditions; solvent effect (PDF)
