# Supplementary material for: Stereochemical Analysis of Trifluoroacetamide Derivatives Based on Through-Space1H–19F Spin–Spin Couplings
Source: J Org Chem. 2023 May 18;88(11):7026–37. doi: 10.1021/acs.joc.3c00311 (PMC10242751; doi:10.1021/acs.joc.3c00311)

Supporting Information

Stereochemical analysis of trifluoroacetamide derivatives based on through-space ^{1}H···^{19}F spin–spin couplings.

Yan Li,[1] Chinatsu Ohtake,[1] Mayuno Hotta,[1] Hidetsugu Tabata,[2] Kiriko Hirano,[3] Motoo Iida,[1] Kayo Nakamura,[1] Kosho Makino,[4] Tetsuta Oshitari,[2] Hideaki Natsugari,[5] Takenori Kusumi,[6] and Hideyo Takahashi*[1]

[1]Faculty of Pharmaceutical Sciences, Tokyo University of Science, 2641Yamazaki, Noda-shi, Chiba 278-8510, Japan
[2]Faculty of Pharma Sciences, Teikyo University, 2-11-1 Kaga, Itabashi-ku, Tokyo 173-8605, Japan
[3]Bruker Japan K.K., 3-9 Moriya, Kanagawa-ku, Yokohama, Kanagawa 221-0022, Japan
[4]Research Institute of Pharmaceutical Sciences, Musashino University, 1-1-20 Shin-machi, Nishitokyo-shi, Tokyo 202-8585, Japan
[5]Graduate School of Pharmaceutical Science, The University of Tokyo, 7-3-1 Hongo, Bunkyo-ku, Tokyo 113-0033, Japan
[6]Department of Chemistry, Tokyo Institute of Technology, Meguro-ku, Tokyo 152-8551, Japan

Corresponding Author
*E-mail: hide-tak@rs.tus.ac.jp

Contents

1. H^{6a} region of the ^{1}H NMR spectrum of 1 .... S3
2. H^{4a} region of the ^{1}H NMR spectrum of 2 .... S3
3. H^{6} and H^{4} regions of the ^{1}H NMR spectra of 1b and 2b, respectively S4
4. ^{1}H-{^{19}F} spectra of 1b and 2b.. S5
5. ^{19}F Spectra of 1a-b and 2a-b... S6
6. ^{1}H-^{19}F HOESY experiments... S8
7. 2D-^{1}H-^{19}F HOESY experiments.. S9
8. X-ray crystal data for 2b.. S10
9. ^{1}H-, ^{13}C-, and 2D-NMR Spectra... S11
10. DFT calculations... S16
11. 1D and 2D HOESY experimental conditions.. S31
12. Solvent effect... S32

1. H^{6a} region of the ^1H NMR spectrum of 1.

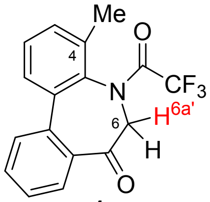

1a

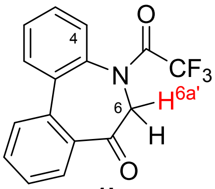

1b

^1H NMR (400 MHz, $CDCl_3$, ppm)

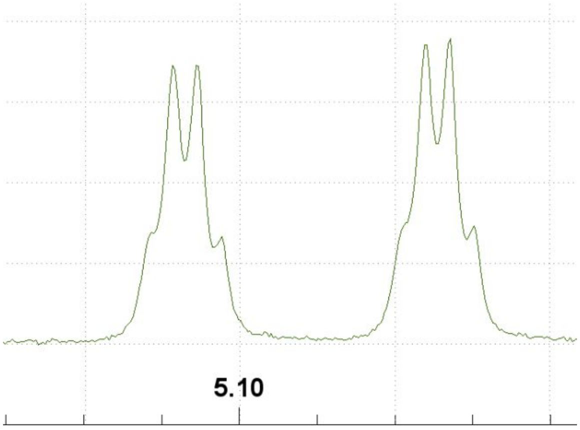

^1H NMR (400 MHz, $CDCl_3$, ppm)

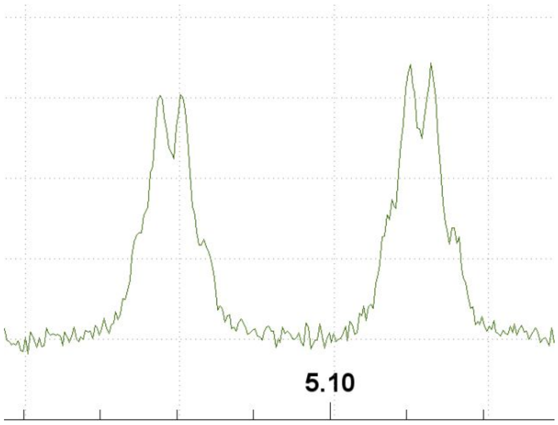

2. H^{4a} region of the ^1H NMR spectrum of 2.

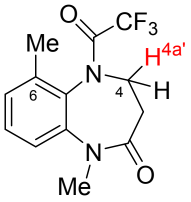

2a

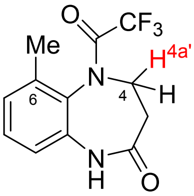

2b

^1H NMR (400 MHz, $CDCl_3$, ppm)

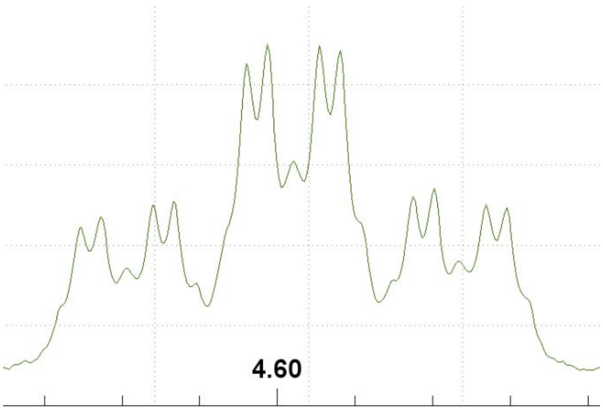

^1H NMR (400 MHz, $CDCl_3$, ppm)

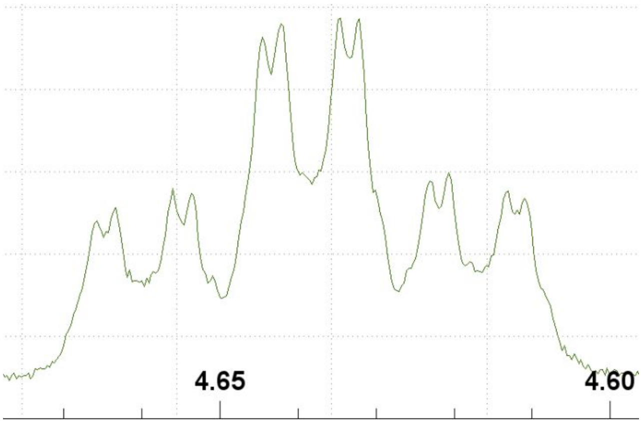

3. H^6 and H^4 regions of the ^1H NMR spectra of 1b and 2b, respectively

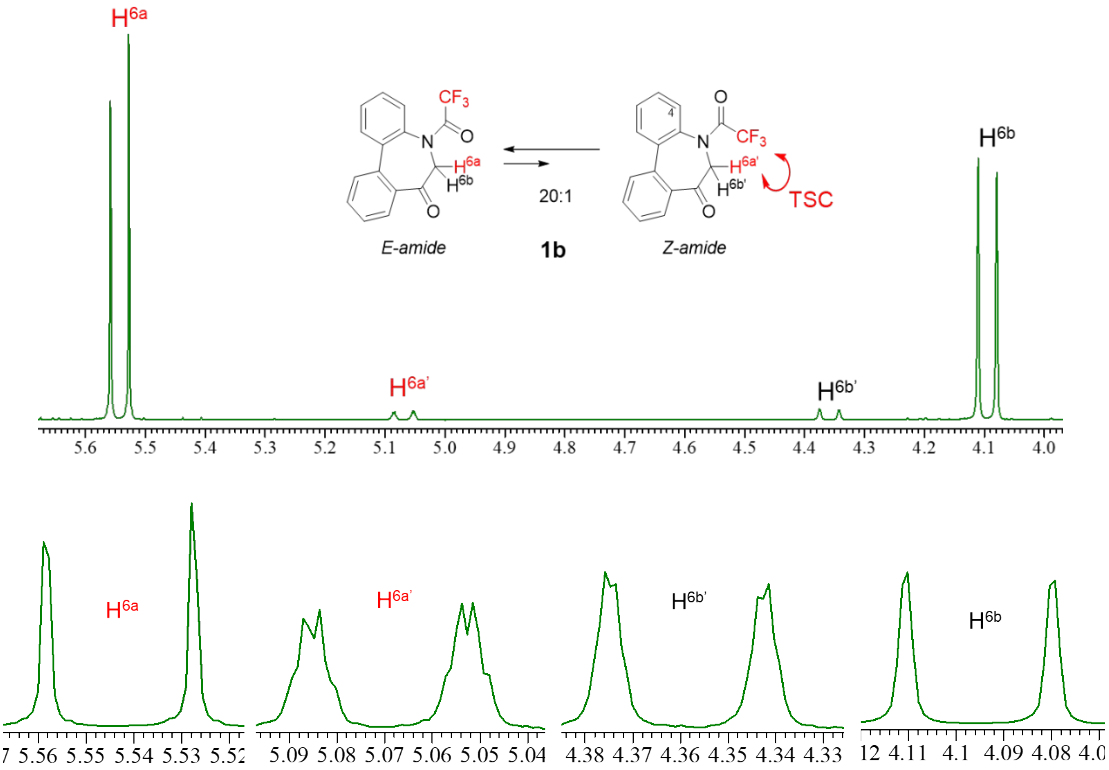

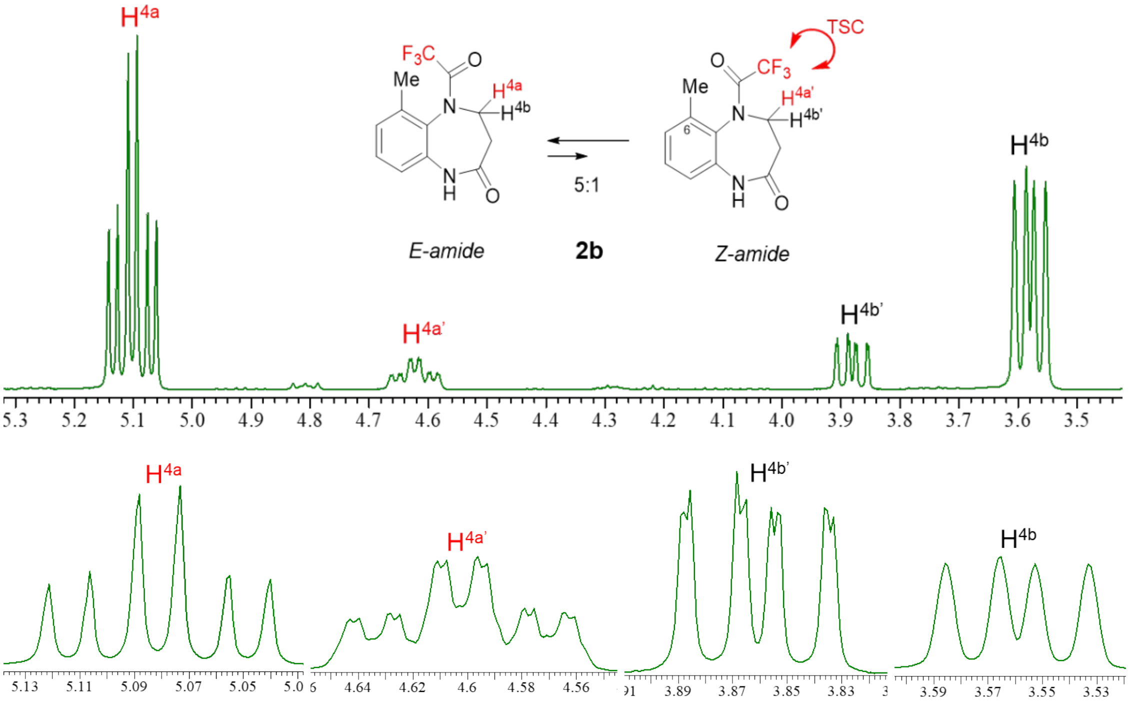

4. ^{1}H-{^{19}F} spectra of 1b and 2b

(1b)

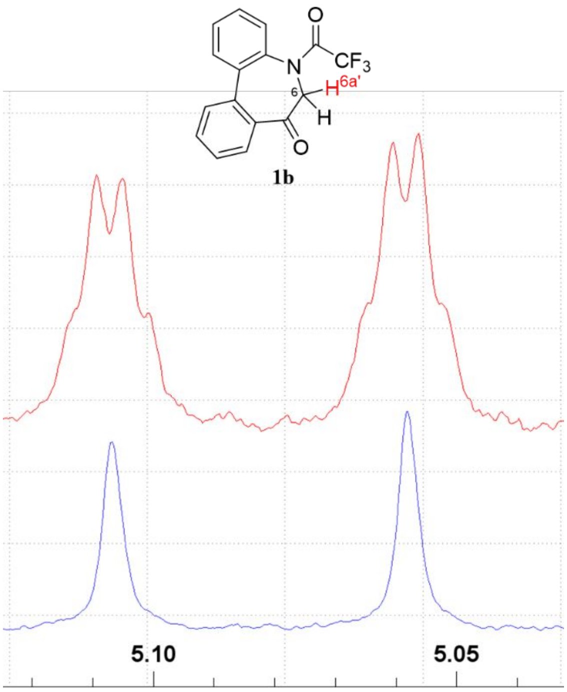

(2b)

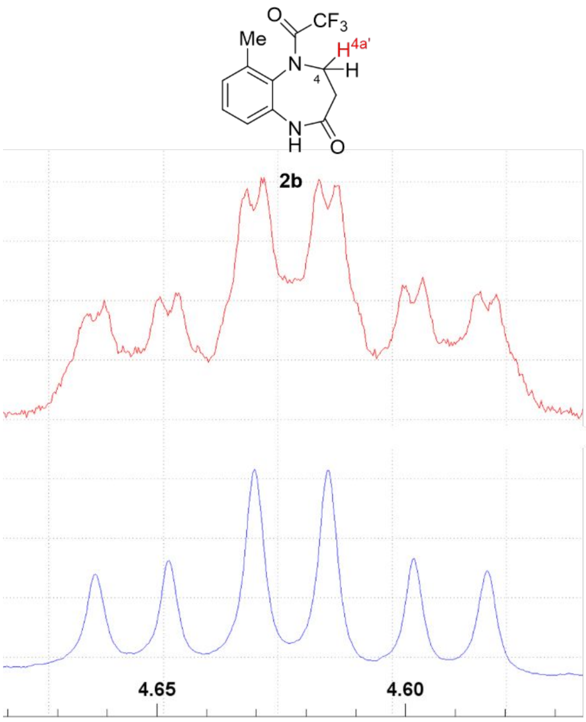

5. ^{19}F Spectra of 1a-b and 2a-b.

(1a)

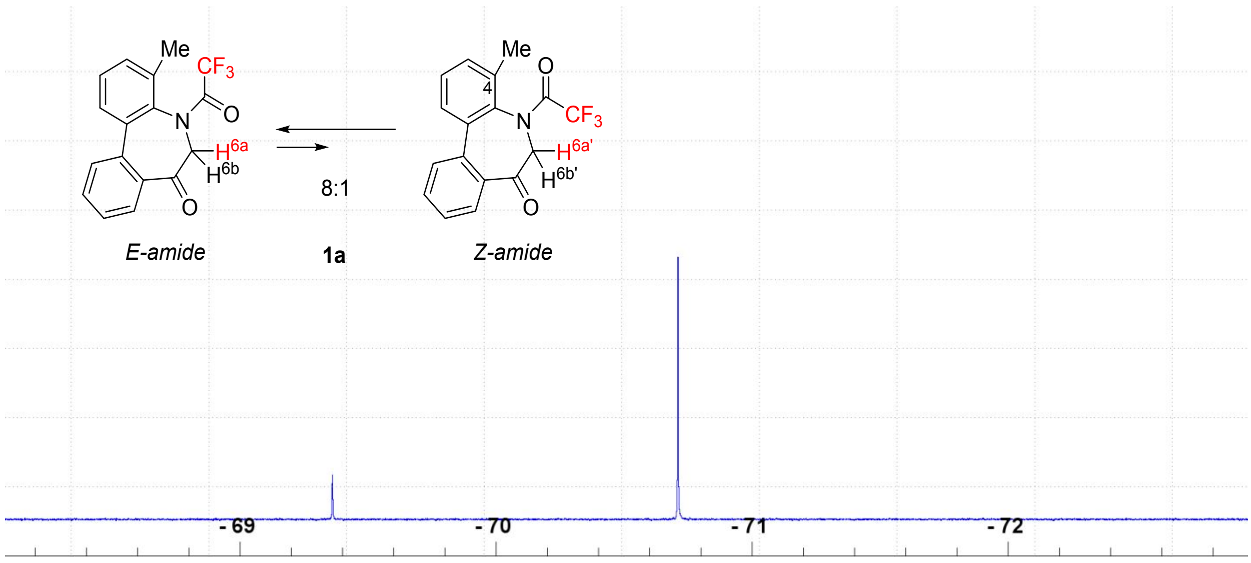

(1b)

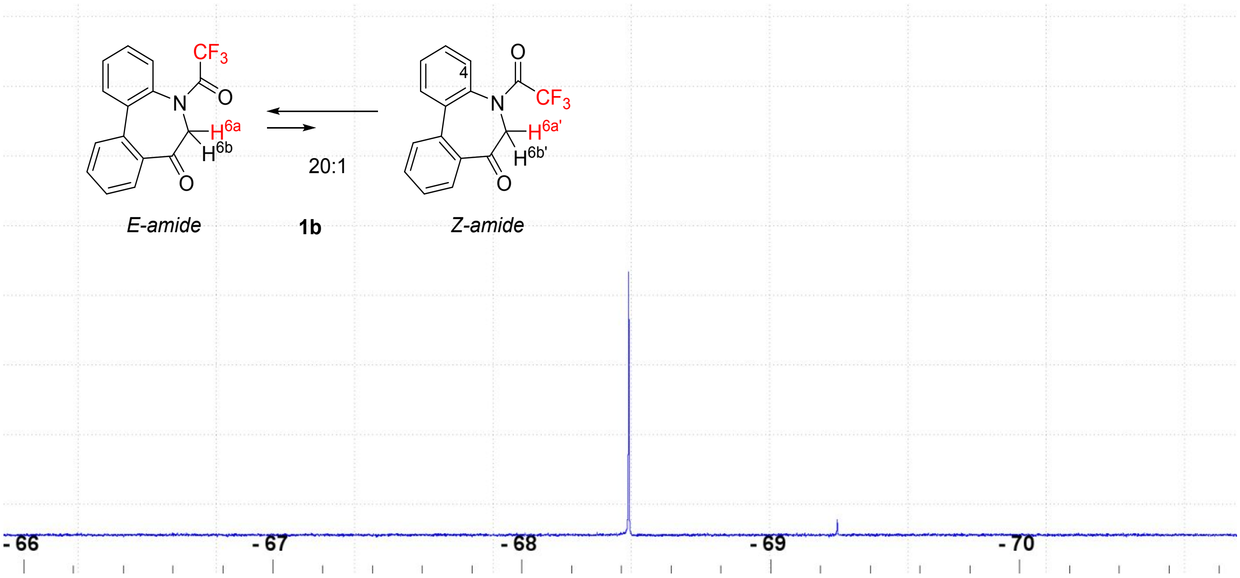

(2a)

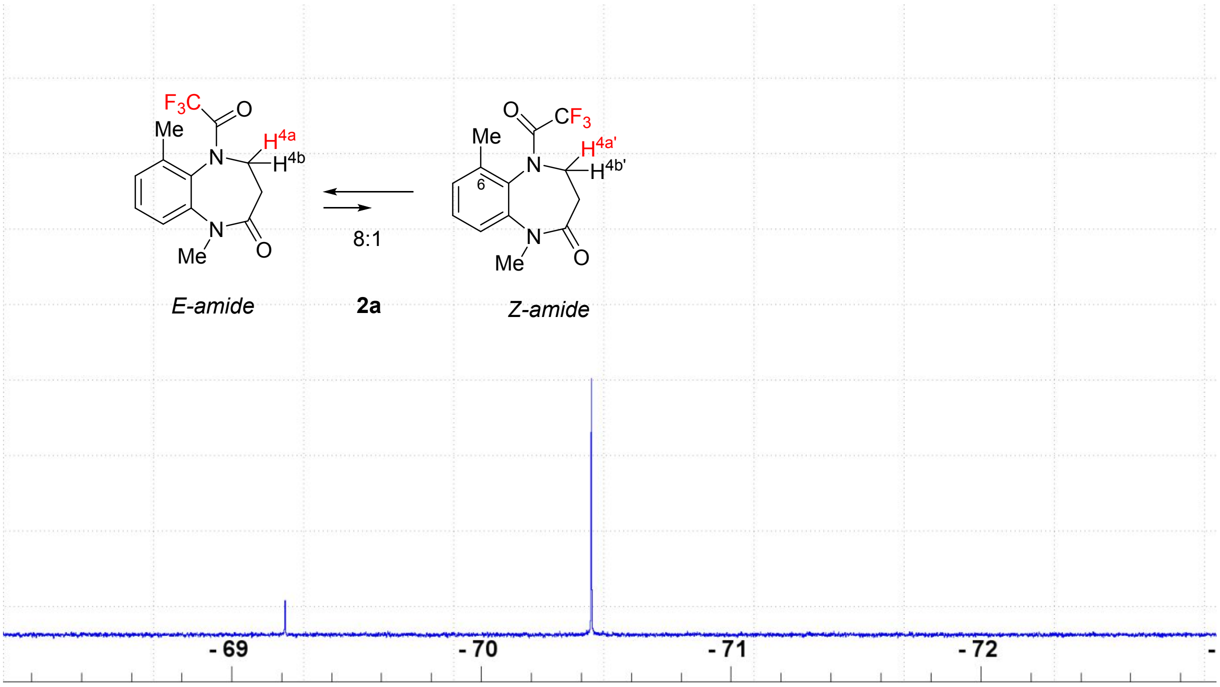
F3C
O
Me
N
H4a
H4b
N
Me
O
8:1
O
CF3
Me
6
N
H4a'
H4b'
N
Me
O
E-amide
2a
Z-amide
- 69
- 70
- 71
- 72

(2b)

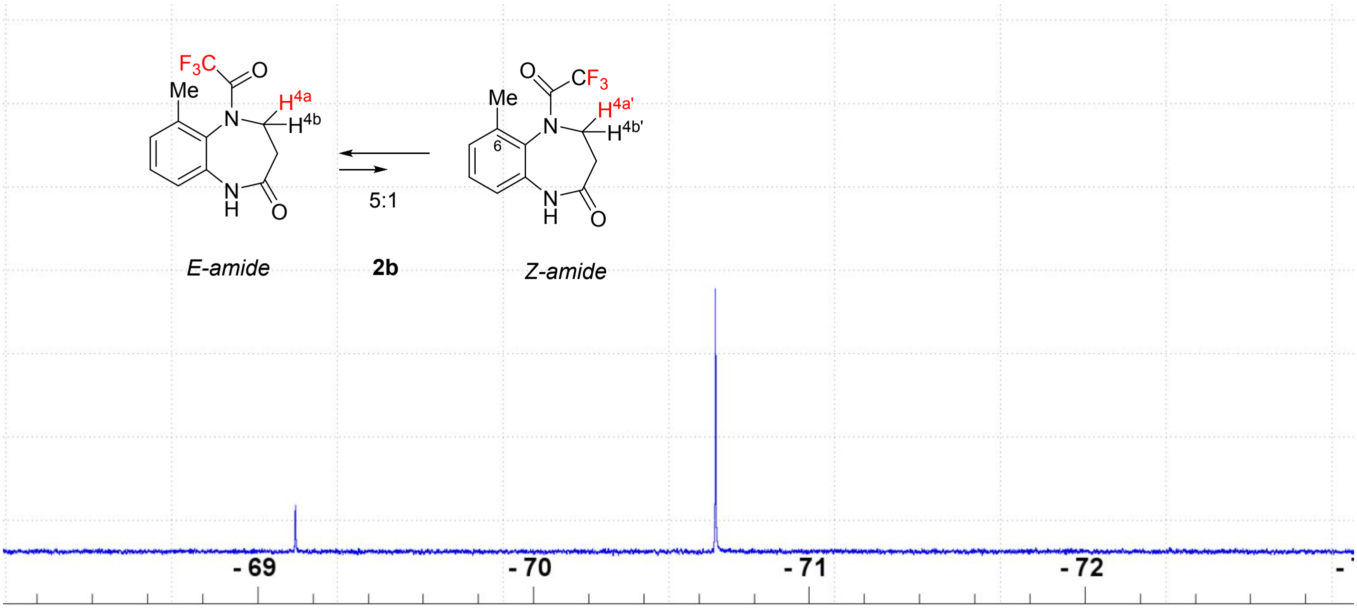
F3C
O
Me
N
H4a
H4b
N
H
O
5:1
O
CF3
Me
6
N
H4a'
H4b'
N
H
O
E-amide
2b
Z-amide
- 69
- 70
- 71
- 72

6. ^{1}H-^{19}F HOESY experiments

(1a)

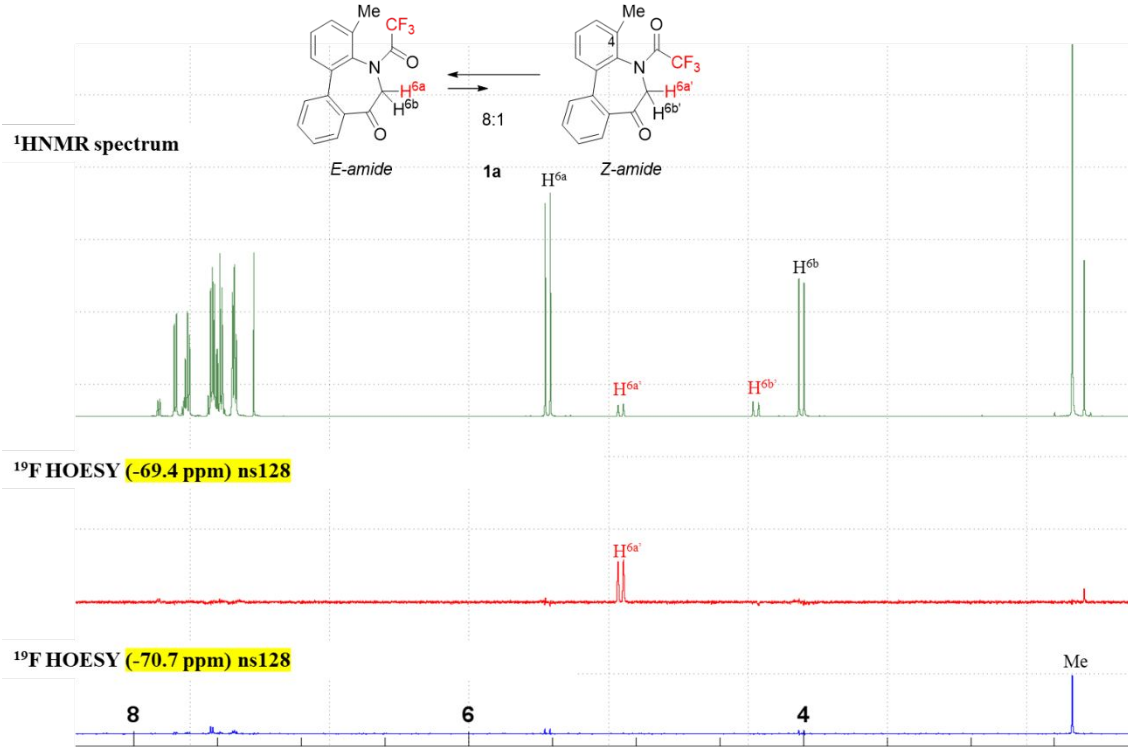

(2a)

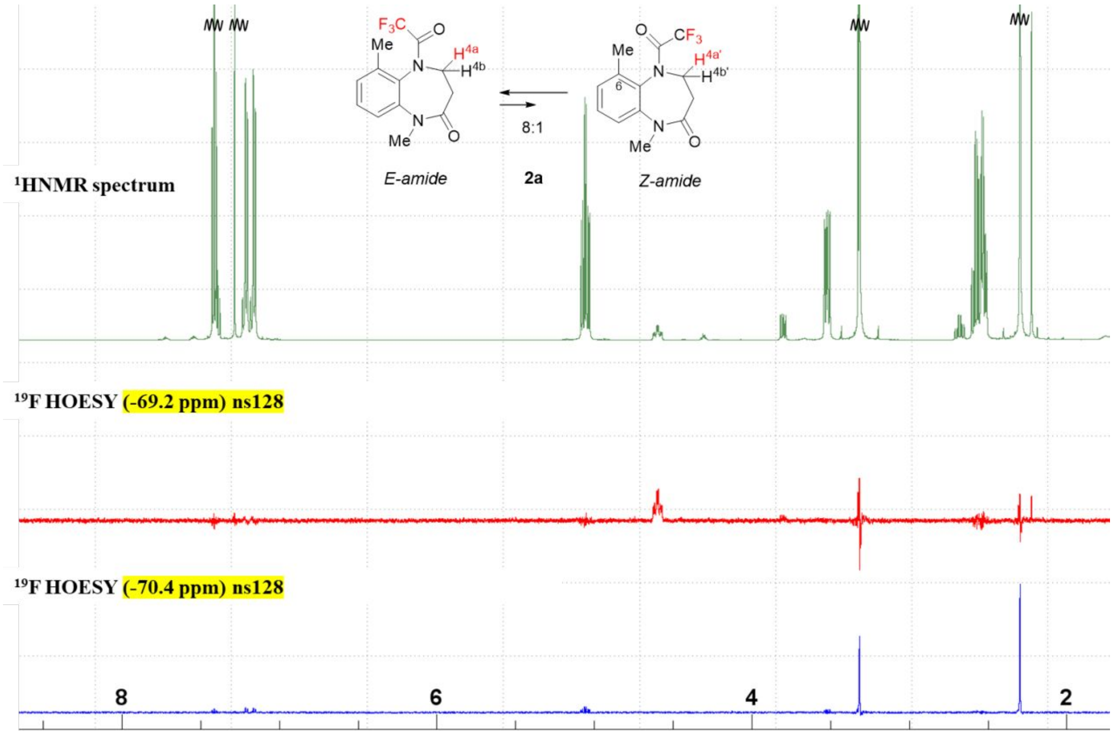

7. 2D ^{1}H-^{19}F HOESY experiments

(2a)

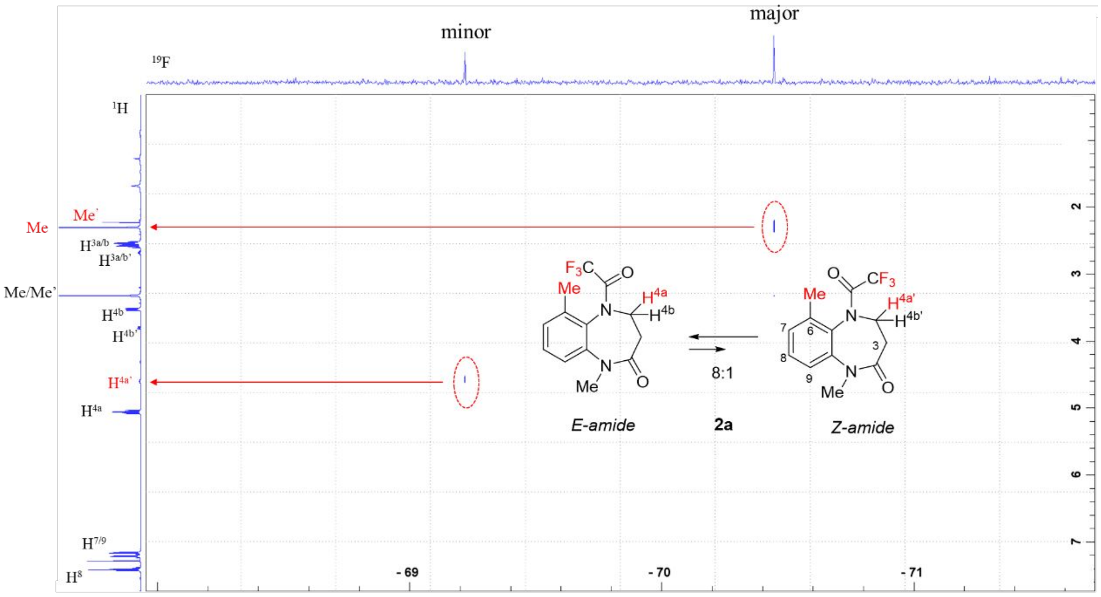

(2b)

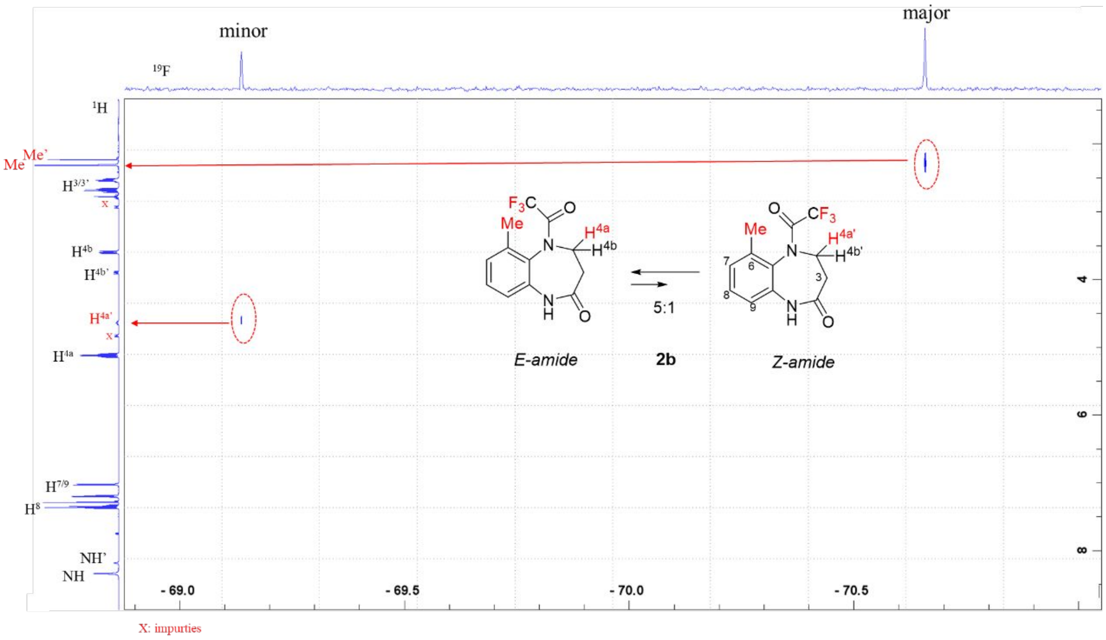

8. X-ray crystal data for 2b

All the measurements were performed using a Rigaku Raxis Rapid imaging plate area detector with graphite monochromated Cu-Kα radiation. The data were collected at a temperature of –100 °C. The structure was solved using direct method SIR97 and expanded using Fourier techniques. The non-hydrogen atoms were refined anisotropically. All calculations were performed using the crystal structure (Crystal Structure 4.2.2) crystallographic software package except for refinement, which was performed using SHELXL97. Typical crystal data and ORTEP diagrams are as follows.
Crystals of **2b** were obtained by dissolving the compounds in diisopropyl ether/hexane and allowing the solvent to slowly evaporate at room temperature.

Crystal data of 2b (CCDC 2239987)

$C_{12}H_{11}F_3N_2O_2$: CuKα (λ = 1.54187 Å), triclinic, $P2_1/c$, colorless block 0.150 × 0.100 × 0.040 mm, crystal dimensions a = 9.25281(17) Å, b = 10.8154(2) Å,
c = 12.4643(2) Å, α = 82.3338(10)°, β = 87.8603(10)°, γ = 83.6129(10)°, T = 173 K, Z = 3, V = 1228.20(4) Å^3, D_{calc} = 1.513 g/cm^3, μ CuKα = 8.804 cm^{-1}, F_{000} = 720.00,
R_{int} = 0.0474, R_1 = 0.0478, wR_2 = 0.1090

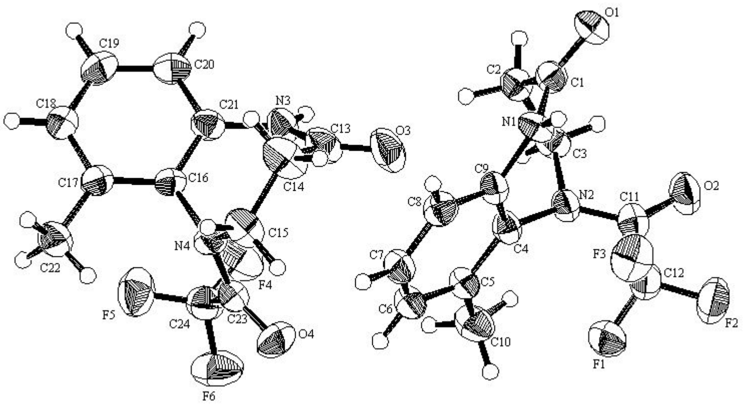

ORTEP view
Ellipsoid contour probability = 50%

9. ¹H-, ¹³C-, and 2D-NMR Spectra

^{13}C-NMR (100 MHz, $CDCl_3$) of **1a**

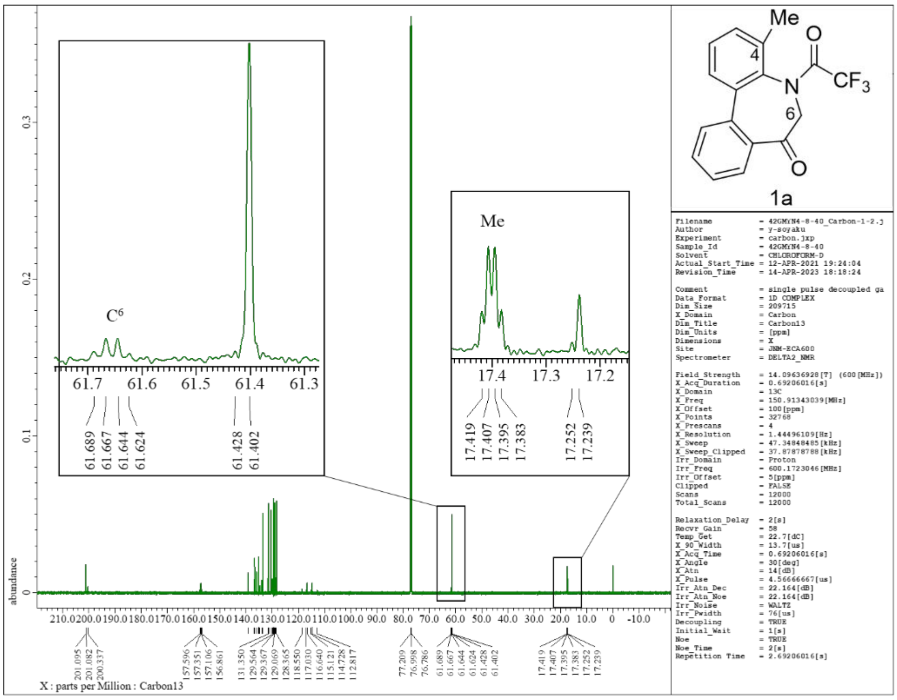

^{1}H-NMR (400 MHz, $CDCl_3$) of **2a**

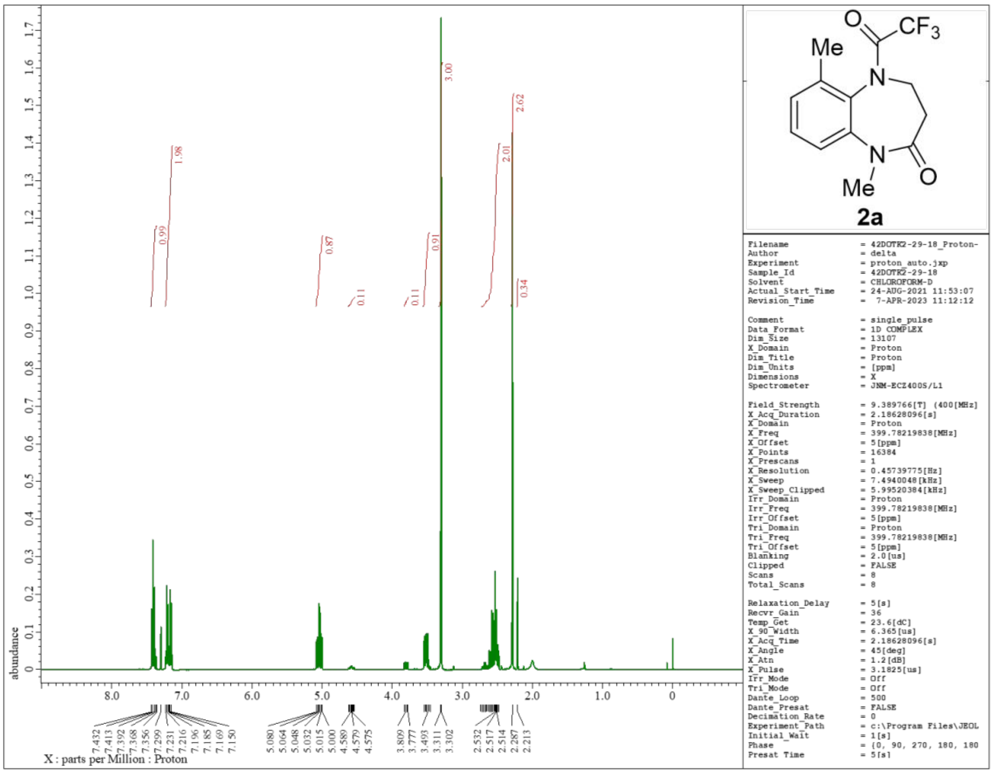

^{13}C-NMR (100 MHz, $CDCl_3$) of **2a**

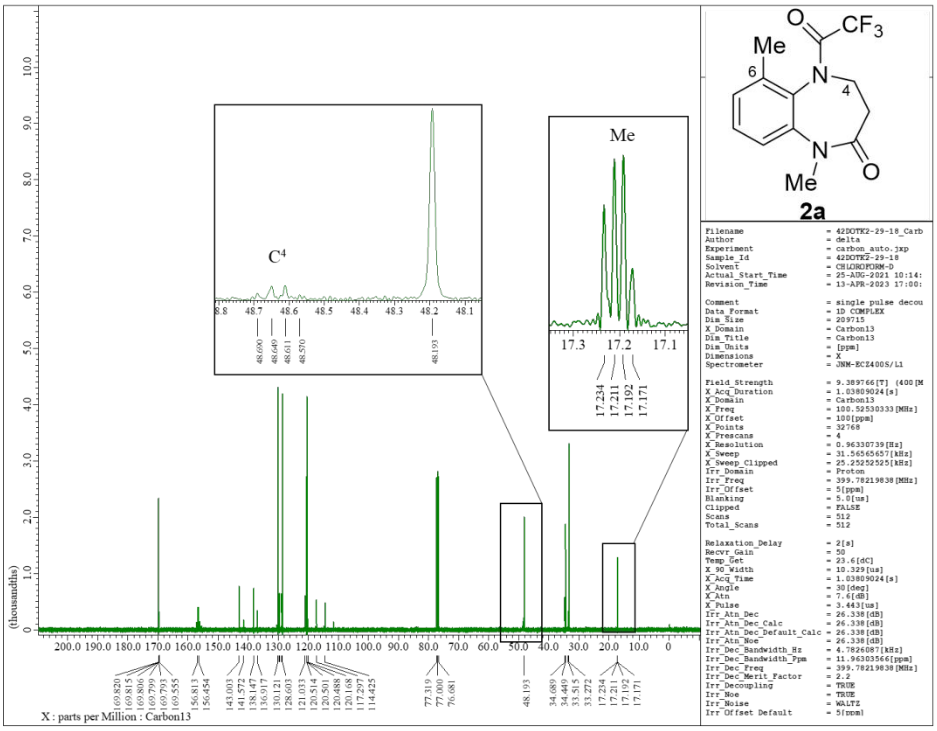

^1H-^1H COSY NMR (400 MHz, $CDCl_3$) of **2a**

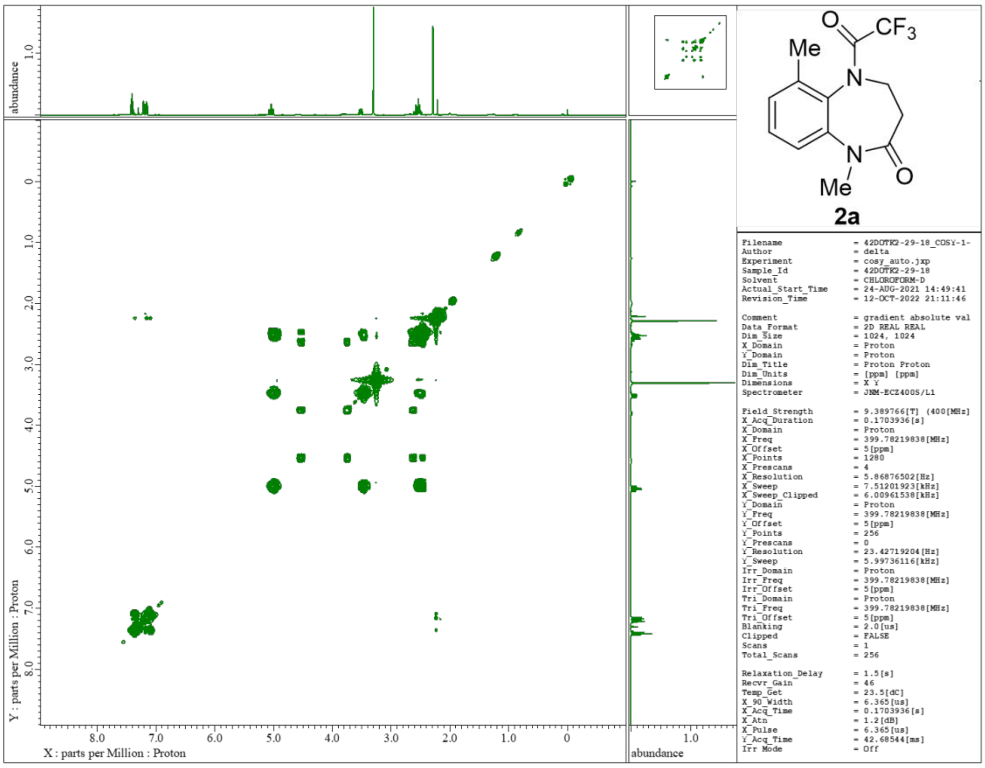

HMQC NMR (400 MHz, $CDCl_3$) of **2a**

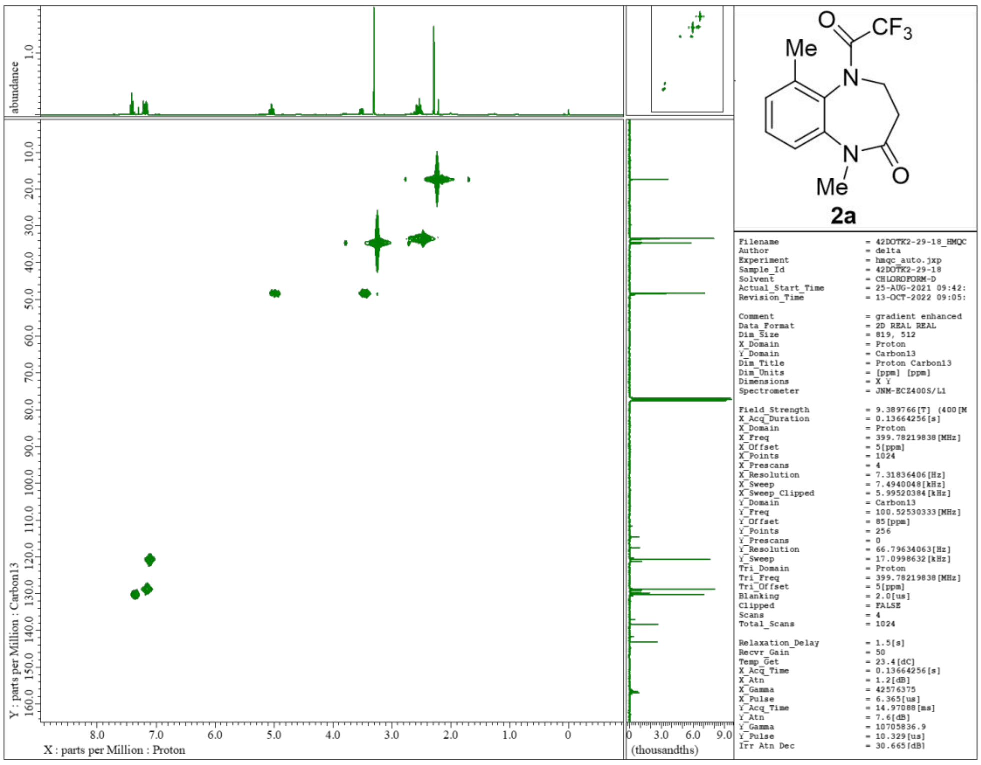

^{1}H-NMR (400 MHz, $CDCl_3$) of **2b**

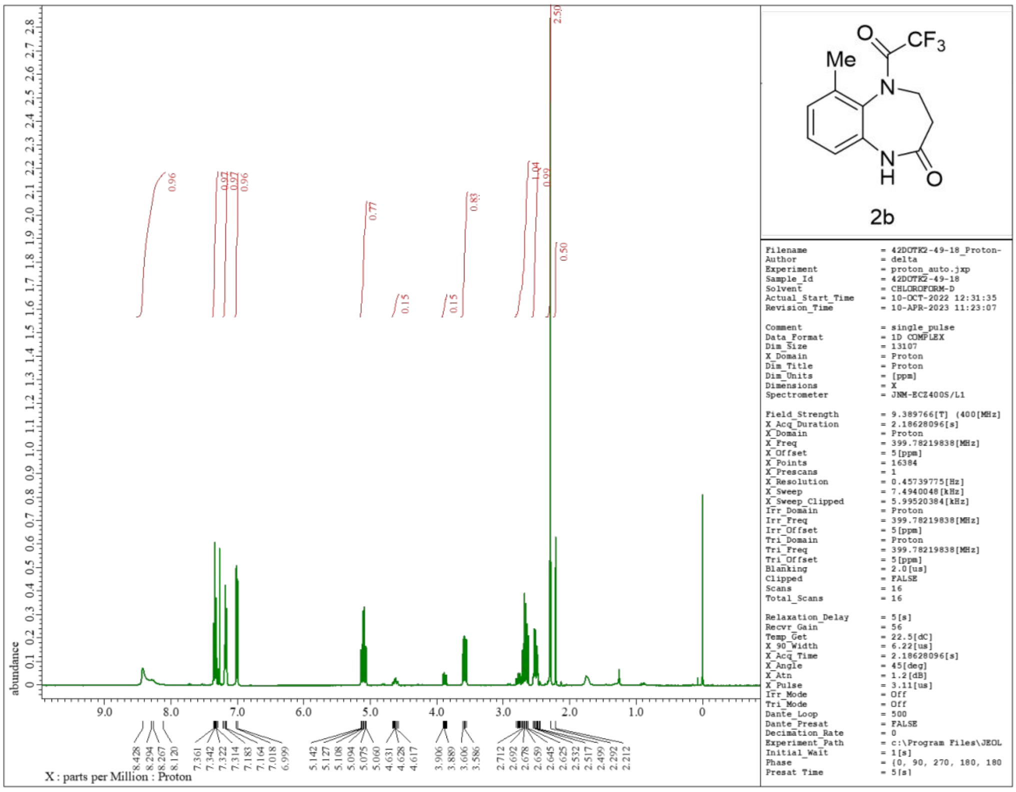

^{13}C-NMR (100 MHz, $CDCl_3$) of **2b**

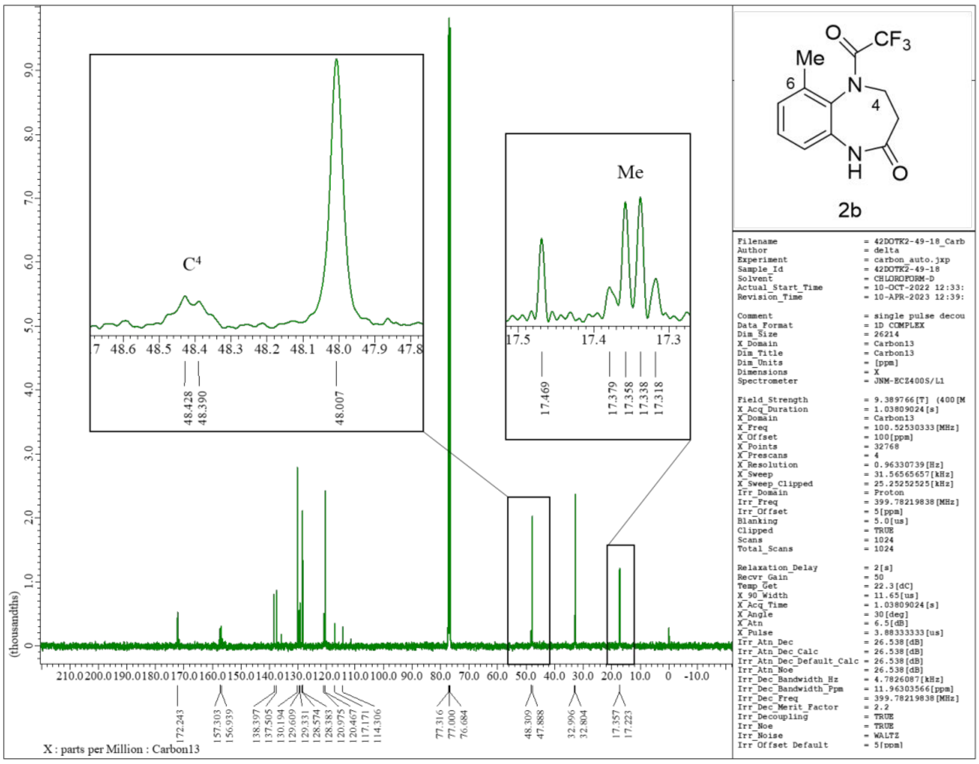

^{1}H-^{1}H COSY NMR (400 MHz, $CDCl_3$) of **2b**

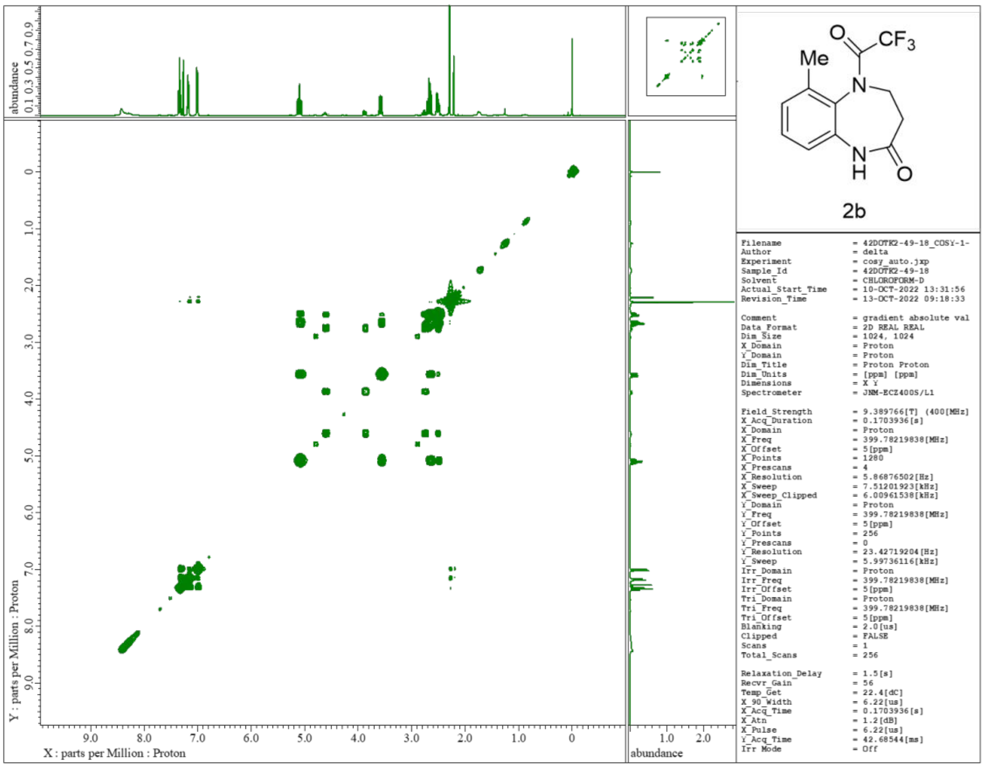

HMQC NMR (400 MHz, $CDCl_3$) of **2b**

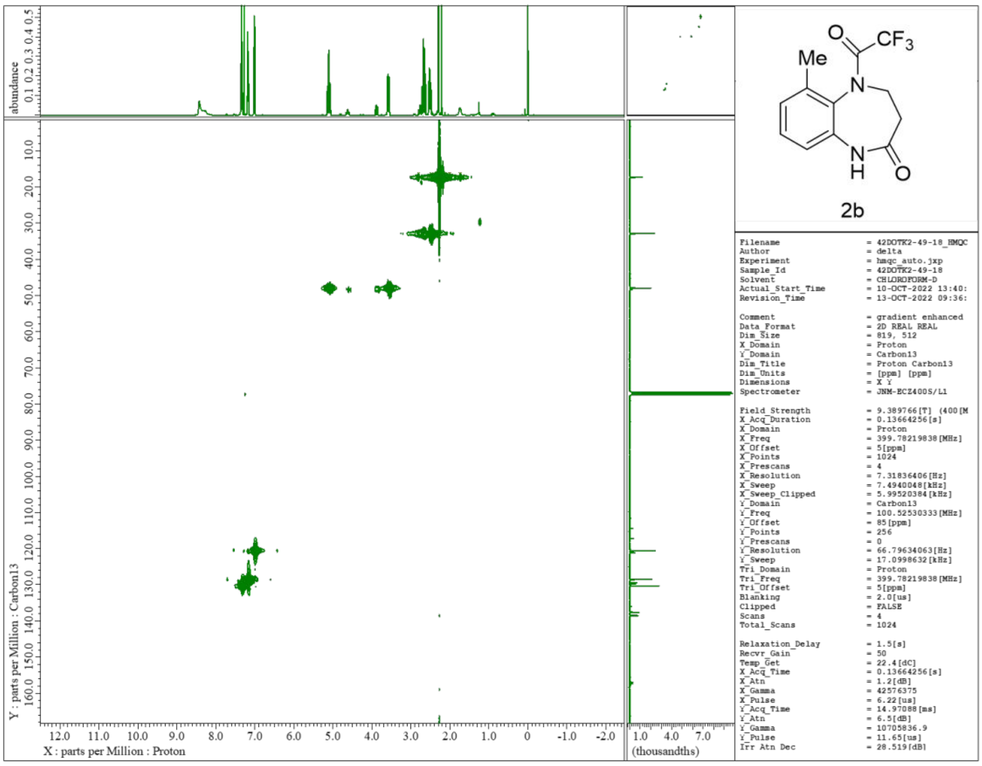

10. DFT calculations

Conformation searches and optimization were conducted with Spartan'20 (Wavefunction, Inc.) using a PC (Windows10 Pro; Intel Xeon Silver 4214R CPU processor, 2.40 GHz, 24 cores; 64 GB RAM).

Table S1. Geometry and energy of **1a** calculated at the B3LYP/6-31G(d), SM8:$CHCl_3$ level

conf ID	M001	M002
Geometry type	*E*	*Z*
SCF (au)	-1159.5740	-1159.5735
rel. SCF (KJ/mol)	0	1.39
Boltzman dist.	63.7%	36.3%

XYZ coordinates of **1a** calculated at the B3LYP/6-31G(d), SM8:$CHCl_3$ level

35

M0001

H	-1.0978	1.3425	-4.0115
C	-0.8560	1.2601	-2.9561v
C	-0.2730	1.0505	-0.2170
C	-0.7920	2.4046	-2.1639
C	-0.6334	-0.0093	-2.4113
C	-0.3111	-0.0852	-1.0455
C	-0.5132	2.3024	-0.8047
H	-0.9804	3.3788	-2.6037
H	-0.5040	3.1908	-0.1828
N	-0.1400	-1.3714	-0.4198
C	-1.2933	-1.8205	0.3554
C	-1.4428	-1.1262	1.7134
C	-0.5225	-0.0122	2.1149
C	0.0315	0.9589	1.2391
H	-2.2031	-1.6549	-0.2289
H	-1.2007	-2.8902	0.5505
C	0.8639	1.9452	1.7953
H	1.3069	2.6834	1.1355
C	1.1490	1.9849	3.1569
H	1.8113	2.7524	3.5448
C	0.5856	1.0397	4.0150

H	0.7960	1.0636	5.0792
C	-0.2497	0.0613	3.4910
H	-0.7112	-0.6781	4.1361
O	-2.2927	-1.5552	2.4814
C	1.0369	-2.0414	-0.2522
C	2.3106	-1.4236	-0.8964
O	1.1536	-3.0804	0.3809
F	3.3596	-2.2188	-0.6644
F	2.5831	-0.2131	-0.3697
F	2.1989	-1.2764	-2.2341
C	-0.7645	-1.2472	-3.2643
H	0.1804	-1.7925	-3.3339
H	-1.5061	-1.9408	-2.8518
H	-1.0795	-0.9813	-4.2766

M0002

H	-0.7313	1.5175	-4.1940
C	-0.5810	1.4446	-3.1209
C	-0.1948	1.2580	-0.3419
C	-0.6168	2.5919	-2.3308
C	-0.3732	0.1841	-2.5526
C	-0.1868	0.1160	-1.1623
C	-0.4247	2.5012	-0.9568
H	-0.8019	3.5591	-2.7872
H	-0.4687	3.3945	-0.3437
N	-0.0679	-1.1654	-0.5182
C	-1.2371	-1.5410	0.2796
C	-1.2934	-0.9156	1.6747
C	-0.4166	0.2416	2.0307
C	0.0939	1.2061	1.1213
H	-2.1291	-1.2304	-0.2742
H	-1.3062	-2.6205	0.4036
C	0.9008	2.2312	1.6490
H	1.3234	2.9616	0.9681
C	1.1940	2.3186	3.0061
H	1.8348	3.1177	3.3653

C	0.6700	1.3794	3.8954
H	0.8884	1.4388	4.9566
C	-0.1322	0.3600	3.4026
H	-0.5618	-0.3810	4.0677
O	-2.0441	-1.4263	2.4947
C	1.1081	-1.8483	-0.6003
C	1.1925	-3.1903	0.1755
O	2.0799	-1.4886	-1.2474
F	2.4207	-3.6995	0.0772
F	0.3211	-4.1019	-0.3144
F	0.9161	-3.0208	1.4898
C	-0.3506	-1.0549	-3.4107
H	0.6564	-1.4847	-3.4498
H	-1.0188	-1.8307	-3.0208
H	-0.6633	-0.8220	-4.4319

Table S2. Geometry and energy of **1b** calculated at the B3LYP/6-31G(d), SM8:$CHCl_3$ level

conf ID	M001	M002	M003	M004
Geometry type	*E*	*Z*	*Z*	*E*
SCF (au)	-1120.2837	-1120.2818	-1120.2817	-1120.2837
rel. SCF (KJ/mol)	0.00	4.94	5.38	-0.03
Boltzman dist.	44.7%	6.0%	5.0%	44.3%

XYZ coordinates of **1b** calculated at the B3LYP/6-31G(d), SM8:$CHCl_3$ level

32

M0001

H	1.3550	-0.5023	-4.3765
C	1.0671	-0.1672	-3.3857
C	0.3253	0.6922	-0.7972
C	0.8801	1.1894	-3.1213
C	0.9014	-1.0960	-2.3603
C	0.5269	-0.6702	-1.0856
C	0.5166	1.6097	-1.8440
H	1.0256	1.9241	-3.9064

H	1.0536	-2.1547	-2.5364
H	0.3916	2.6680	-1.6416
N	0.4504	-1.6160	-0.0061
C	1.5360	-1.5177	0.9665
C	1.4114	-0.3569	1.9568
C	0.3667	0.7027	1.7819
C	-0.1335	1.1690	0.5378
H	1.5697	-2.4369	1.5531
H	2.4850	-1.4189	0.4306
C	-1.0941	2.1953	0.5609
C	-1.5537	2.7469	1.7529
C	-1.0453	2.2952	2.9715
C	-0.0871	1.2899	2.9747
H	-1.4989	2.5489	-0.3812
H	-2.3095	3.5255	1.7278
H	-1.3930	2.7201	3.9074
H	0.3360	0.9291	3.9056
C	-0.5687	-2.4839	0.2550
C	-1.7398	-2.5282	-0.7627
O	-0.6051	-3.2354	1.2185
F	-2.7099	-3.3206	-0.2971
F	-1.3374	-3.0267	-1.9520
F	-2.2703	-1.3094	-0.9803
O	2.1482	-0.3649	2.9335

M0002

H	1.0995	-0.2926	-4.6633
C	0.8382	0.0206	-3.6579
C	0.1482	0.8239	-1.0347
C	0.6153	1.3678	-3.3690
C	0.7426	-0.9231	-2.6389
C	0.4098	-0.5215	-1.3458
C	0.2752	1.7600	-2.0775
H	0.7106	2.1164	-4.1489
H	0.9247	-1.9752	-2.8297
H	0.1167	2.8108	-1.8607

N	0.4076	-1.4873	-0.2830
C	1.4924	-1.3132	0.6832
C	1.2488	-0.2334	1.7390
C	0.1834	0.8025	1.5549
C	-0.3189	1.2682	0.3099
H	1.7092	-2.2414	1.2090
H	2.3970	-1.0503	0.1248
C	-1.3149	2.2616	0.3407
C	-1.8005	2.7857	1.5343
C	-1.2877	2.3375	2.7523
C	-0.3007	1.3617	2.7504
H	-1.7295	2.6098	-0.5988
H	-2.5818	3.5389	1.5106
H	-1.6559	2.7400	3.6902
H	0.1246	0.9997	3.6798
C	-0.6079	-2.3941	-0.2085
C	-0.5743	-3.3756	0.9947
O	-1.5220	-2.4868	-1.0121
F	-1.6593	-4.1501	0.9856
F	-0.5420	-2.7155	2.1751
F	0.5133	-4.1786	0.9452
O	1.9382	-0.2664	2.7491

M0003

H	1.1716	-0.1664	-4.6437
C	0.9029	0.1206	-3.6325
C	0.1907	0.8553	-0.9937
C	0.7204	1.4641	-3.2999
C	0.7602	-0.8533	-2.6486
C	0.4183	-0.4858	-1.3472
C	0.3700	1.8220	-2.0018
H	0.8551	2.2356	-4.0510
H	0.9138	-1.9032	-2.8721
H	0.2440	2.8695	-1.7508
N	0.3766	-1.4836	-0.3164
C	1.4381	-1.3508	0.6844

C	1.1495	-0.3395	1.7930
C	0.1379	0.7470	1.5992
C	-0.2968	1.2735	0.3521
H	1.6617	-2.3047	1.1586
H	2.3474	-1.0369	0.1611
C	-1.2510	2.3086	0.3824
C	-1.7557	2.8179	1.5739
C	-1.3078	2.3097	2.7942
C	-0.3678	1.2894	2.7940
H	-1.6195	2.7015	-0.5587
H	-2.5015	3.6061	1.5466
H	-1.6910	2.6995	3.7315
H	0.0059	0.8774	3.7247
C	-0.6263	-2.4075	-0.3182
C	-0.6240	-3.4388	0.8427
O	-1.5095	-2.4778	-1.1583
F	-1.7028	-4.2176	0.7626
F	-0.6360	-2.8310	2.0506
F	0.4689	-4.2352	0.7931
O	1.7565	-0.4655	2.8481

M0004

H	1.5572	-0.4874	-4.3123
C	1.2328	-0.1572	-3.3311
C	0.3979	0.6898	-0.7653
C	1.0960	1.2016	-3.0482
C	0.9717	-1.0952	-2.3346
C	0.5520	-0.6751	-1.0719
C	0.6864	1.6155	-1.7829
H	1.3171	1.9431	-3.8090
H	1.0858	-2.1564	-2.5237
H	0.6013	2.6751	-1.5670
N	0.3860	-1.6341	-0.0149
C	1.4236	-1.5883	1.0124
C	1.2510	-0.4702	2.0422
C	0.2846	0.6521	1.8162

C	-0.1105	1.1661	0.5523
H	1.4269	-2.5319	1.5590
H	2.3960	-1.4695	0.5245
C	-1.0149	2.2434	0.5393
C	-1.5154	2.8020	1.7110
C	-1.1080	2.3044	2.9494
C	-0.2105	1.2459	2.9901
H	-1.3452	2.6328	-0.4175
H	-2.2245	3.6218	1.6543
H	-1.4877	2.7334	3.8708
H	0.1328	0.8451	3.9372
C	-0.6710	-2.4736	0.1779
C	-1.7949	-2.4573	-0.8926
O	-0.7777	-3.2415	1.1234
F	-2.8164	-3.2140	-0.4796
F	-1.3598	-2.9597	-2.0683
F	-2.2641	-1.2144	-1.1174
O	1.9017	-0.5464	3.0760

Table S3. Geometry and energy of **2a** calculated at the B3LYP/6-31G(d), SM8:$CHCl_3$ level

conf ID	M001	M002	M003	M004
Geometry type	*E*	*Z*	*E*	*Z*
SCF (au)	-1062.5138	-1062.5124	-1062.5136	-1062.5125
rel. SCF (KJ/mol)	0.00	3.57	0.47	3.33
Boltzman dist.	43.1%	10.2%	35.6%	11.2%

XYZ coordinates of **2a** calculated at the B3LYP/6-31G(d), SM8:$CHCl_3$ level

33

M0001

C	0.0627	1.6260	-1.1505
C	0.6358	1.6998	1.6136
C	0.3142	2.8375	-0.4959
C	0.0609	0.4505	-0.3810
C	0.4024	0.4726	0.9857

C	0.5780	2.8768	0.8720
H	0.8784	1.7228	2.6702
H	0.3035	3.7561	-1.0745
H	0.7658	3.8277	1.3602
N	0.4815	-0.7431	1.7204
C	1.2197	-1.8278	1.2808
C	1.9420	-1.6554	-0.0493
C	0.9884	-1.6674	-1.2573
N	-0.1975	-0.8272	-0.9923
H	2.5192	-0.7259	-0.0349
H	2.6412	-2.4880	-0.1444
H	0.6227	-2.6729	-1.4640
H	1.5078	-1.2984	-2.1474
C	-1.4291	-1.3912	-1.1412
C	-2.6552	-0.5976	-0.6024
O	-1.6374	-2.4883	-1.6410
F	-2.8197	0.5938	-1.2146
F	-3.7693	-1.3086	-0.8021
F	-2.5474	-0.3697	0.7246
O	1.2980	-2.8651	1.9363
C	-0.1580	-0.8085	3.0374
H	-0.3178	-1.8574	3.2845
H	-1.1144	-0.2847	2.9986
H	0.4707	-0.3629	3.8162
C	-0.2083	1.5984	-2.6351
H	-0.0064	2.5775	-3.0766
H	-1.2500	1.3404	-2.8481
H	0.4177	0.8602	-3.1479

M0002

C	0.4521	1.8633	-0.7775
C	0.0754	1.7690	2.0142
C	0.5695	3.0166	0.0065
C	0.1216	0.6564	-0.1379
C	-0.0421	0.5973	1.2594
C	0.3717	2.9742	1.3847

H	-0.0551	1.7244	3.0900
H	0.8185	3.9551	-0.4794
H	0.4666	3.8796	1.9756
N	-0.3517	-0.6343	1.9019
C	0.3766	-1.7861	1.6844
C	1.5069	-1.6817	0.6716
C	1.0029	-1.5862	-0.7750
N	-0.0501	-0.5520	-0.9028
H	2.1417	-0.8222	0.9070
H	2.1093	-2.5865	0.7691
H	0.5898	-2.5469	-1.0720
H	1.8381	-1.3484	-1.4425
C	-1.1561	-0.6455	-1.6940
C	-1.2817	-1.9051	-2.5960
O	-2.0452	0.1916	-1.7580
F	-2.3243	-1.7711	-3.4184
F	-0.1784	-2.0921	-3.3530
F	-1.4766	-3.0273	-1.8630
O	0.1279	-2.8311	2.2833
C	-1.3769	-0.6485	2.9506
H	-1.8161	-1.6450	2.9893
H	-2.1444	0.0873	2.7084
H	-0.9531	-0.4233	3.9357
C	0.6386	1.9385	-2.2717
H	-0.3310	1.9677	-2.7829
H	1.1831	1.0723	-2.6613
H	1.1925	2.8402	-2.5460

M0003

C	-0.5866	1.6564	-0.9437
C	-0.9002	1.2974	1.8394
C	-1.0835	2.6839	-0.1335
C	-0.2101	0.4529	-0.3236
C	-0.4150	0.2471	1.0543
C	-1.2208	2.5138	1.2430
H	-1.0398	1.1470	2.9042

H	-1.3665	3.6246	-0.5958
H	-1.6044	3.3253	1.8530
N	-0.1095	-1.0124	1.6390
C	-0.5880	-2.2035	1.1230
C	-1.4349	-2.1027	-0.1397
C	-0.6123	-1.7457	-1.3903
N	0.3202	-0.6377	-1.0995
H	-1.9002	-3.0781	-0.2914
H	-2.2283	-1.3640	0.0100
H	-1.2834	-1.4621	-2.2080
H	-0.0070	-2.5885	-1.7233
C	1.6379	-0.8187	-1.3958
C	2.6576	0.2086	-0.8222
O	2.0817	-1.7510	-2.0520
F	2.6092	0.2503	0.5270
F	3.8957	-0.1550	-1.1721
F	2.4576	1.4617	-1.2811
O	-0.3553	-3.2800	1.6700
C	0.6091	-1.0443	2.9162
H	1.1323	-1.9971	2.9912
H	-0.0741	-0.9611	3.7690
H	1.3273	-0.2238	2.9433
C	-0.4531	1.8500	-2.4349
H	-0.9079	1.0240	-2.9917
H	0.5962	1.9063	-2.7408
H	-0.9441	2.7765	-2.7432

M0004

C	0.1769	1.6995	-1.1837
C	0.4721	2.1028	1.5912
C	0.4294	2.9730	-0.6618
C	0.0582	0.6265	-0.2845
C	0.2295	0.8154	1.0998
C	0.5638	3.1759	0.7101
H	0.5991	2.2504	2.6580
H	0.5171	3.8110	-1.3465

H	0.7571	4.1720	1.0951
N	0.1309	-0.2832	1.9998
C	0.8304	-1.4596	1.8129
C	1.6969	-1.5290	0.5645
C	0.8734	-1.6844	-0.7218
N	-0.2320	-0.7008	-0.7653
H	2.3301	-0.6391	0.5003
H	2.3451	-2.4005	0.6708
H	0.4559	-2.6872	-0.7615
H	1.5223	-1.5476	-1.5933
C	-1.4949	-0.9453	-1.2139
C	-1.7863	-2.3597	-1.7885
O	-2.4105	-0.1345	-1.2063
F	-3.0144	-2.3928	-2.3101
F	-0.9135	-2.6994	-2.7618
F	-1.7210	-3.3096	-0.8252
O	0.7669	-2.3874	2.6175
C	-0.6166	-0.1075	3.2491
H	-1.4842	0.5277	3.0646
H	0.0032	0.3436	4.0324
H	-0.9413	-1.0886	3.5939
C	0.0019	1.5021	-2.6682
H	0.3571	2.3781	-3.2169
H	-1.0544	1.3505	-2.9190
H	0.5516	0.6275	-3.0319

Table S4. Geometry and energy of **2b** calculated at the B3LYP/6-31G(d), SM8:$CHCl_3$ level

conf ID	M001	M002	M003	M004
Geometry type	*E*	*Z*	*E*	*Z*
SCF (au)	-1023.2306	-1023.2310	-1023.2332	-1023.2328
rel. SCF (KJ/mol)	0.00	-1.02	-6.90	-5.62
Boltzman dist.	3.5%	5.3%	57.2%	34.0%

XYZ coordinates of **2b** calculated at the B3LYP/6-31G(d), SM8:$CHCl_3$ level

M0001

H	1.1598	1.1932	-3.1684
C	0.6440	1.3383	-2.2237
C	-0.7490	1.6877	0.1947
C	0.1337	2.5857	-1.8827
C	0.4821	0.2484	-1.3591
C	-0.1676	0.4471	-0.1252
C	-0.5721	2.7544	-0.6953
H	0.2644	3.4213	-2.5628
H	-1.0086	3.7181	-0.4507
N	-0.3267	-0.6913	0.7329
C	-1.1596	-1.7771	0.1806
C	-0.2999	-2.8005	-0.5569
C	0.7162	-2.3238	-1.5844
N	1.0243	-0.9863	-1.7733
H	-1.8974	-1.3121	-0.4741
H	-1.6849	-2.2703	1.0007
H	-0.9399	-3.5225	-1.0739
H	0.2735	-3.3775	0.1769
H	1.6922	-0.8966	-2.5357
O	1.3219	-3.1570	-2.2548
C	-1.5631	1.8995	1.4487
H	-1.8604	0.9556	1.9104
H	-2.4697	2.4662	1.2149
H	-1.0046	2.4704	2.1972
C	0.4290	-0.9898	1.8262
O	0.3004	-2.0140	2.4843
C	1.5483	0.0093	2.2383
F	2.2638	-0.5194	3.2377
F	1.0598	1.1899	2.6664
F	2.3900	0.2530	1.2113

M0002

H	1.2645	1.4748	-3.3109
C	0.7850	1.6051	-2.3452
C	-0.4828	1.9192	0.1376
C	0.3094	2.8503	-1.9596

C	0.6499	0.4937	-1.4991
C	0.0341	0.6649	-0.2459
C	-0.3280	3.0034	-0.7307
H	0.4216	3.6986	-2.6268
H	-0.7212	3.9708	-0.4343
N	-0.1401	-0.4715	0.6122
C	-0.8811	-1.6040	0.0189
C	0.0339	-2.5829	-0.7091
C	0.9707	-2.0651	-1.7851
N	1.2038	-0.7167	-1.9719
H	-1.6193	-1.1696	-0.6575
H	-1.4349	-2.1320	0.7948
H	-0.5703	-3.3614	-1.1854
H	0.6697	-3.0962	0.0189
H	1.8334	-0.5859	-2.7605
O	1.5749	-2.8684	-2.4940
C	-1.1808	2.1163	1.4594
H	-1.8108	1.2602	1.7219
H	-1.8115	3.0084	1.4292
H	-0.4535	2.2389	2.2700
C	0.4726	-0.4793	1.8318
O	1.2483	0.3750	2.2342
C	0.0838	-1.6225	2.8129
F	0.7088	-1.4523	3.9787
F	0.4177	-2.8479	2.3456
F	-1.2479	-1.6238	3.0499

M0003

H	1.3845	1.0856	-3.0682
C	0.7606	1.2061	-2.1882
C	-0.9204	1.4666	0.0674
C	0.0251	2.3716	-1.9859
C	0.6710	0.1648	-1.2627
C	-0.1232	0.3237	-0.1082
C	-0.8227	2.4879	-0.8875
H	0.0905	3.1795	-2.7075

H	-1.4246	3.3826	-0.7591
N	-0.1317	-0.7507	0.8518
C	-0.8885	-1.9668	0.4911
C	-0.6173	-2.3809	-0.9657
C	0.8490	-2.3126	-1.3669
N	1.3768	-1.0405	-1.4698
H	-1.9599	-1.7880	0.6303
H	-0.5791	-2.7456	1.1876
H	-1.1929	-1.7446	-1.6464
H	-0.9457	-3.4121	-1.1061
H	2.2899	-0.9889	-1.9125
O	1.5263	-3.3031	-1.6226
C	-1.8444	1.6285	1.2498
H	-2.1440	0.6651	1.6705
H	-2.7468	2.1717	0.9549
H	-1.3650	2.1982	2.0530
C	0.6441	-0.7978	1.9724
O	0.6389	-1.7139	2.7836
C	1.6278	0.3822	2.2204
F	2.3543	0.1330	3.3158
F	0.9857	1.5532	2.4183
F	2.4819	0.5451	1.1906

M0004

H	1.5904	1.3273	-3.2000
C	0.9675	1.4563	-2.3203
C	-0.6899	1.7428	-0.0576
C	0.2737	2.6424	-2.1029
C	0.8476	0.4038	-1.4082
C	0.0445	0.5622	-0.2636
C	-0.5591	2.7748	-0.9937
H	0.3660	3.4577	-2.8132
H	-1.1188	3.6925	-0.8401
N	0.0069	-0.5038	0.7060
C	-0.6916	-1.7574	0.3365
C	-0.4505	-2.1294	-1.1326

C	1.0112	-2.0717	-1.5472
N	1.5438	-0.8048	-1.6344
H	-1.7669	-1.6461	0.5123
H	-0.3248	-2.5599	0.9716
H	-1.0320	-1.4701	-1.7851
H	-0.7939	-3.1532	-1.2899
H	2.4612	-0.7496	-2.0686
O	1.6770	-3.0669	-1.8162
C	-1.5628	1.9271	1.1574
H	-2.1023	1.0109	1.4179
H	-2.2965	2.7194	0.9879
H	-0.9582	2.2048	2.0289
C	0.6043	-0.2696	1.9099
O	1.2214	0.7449	2.2003
C	0.4638	-1.3666	3.0027
F	0.9559	-0.9192	4.1597
F	1.1406	-2.4934	2.6763
F	-0.8285	-1.7052	3.2062

11. 1D and 2D HOESY experimental conditions.

All experimental spectra were recorded at a nominal temperature of 298 K on a Bruker AVANCE NEO 400 MHz and 600 MHz spectrometer with a 5 mm BBO probe equipped with a z-gradient coil. (Bruker Corporation, Billerica, MA, USA)

The 1D-HOESY experiment performed at 400 MHz (^{1}H reference frequency: 400.13 MHz, ^{19}F reference frequency: 376.50 MHz) was performed with a gradient selective spin-echo type experiment with a mixing time (D8) of 1 s. The number of scans was set to 128, the sampling point (TD) was set to 65536, the spectra width (SW) was set to 20 ppm, the ^{1}H offset (O1P) and ^{19}F offset were set to 6.2 ppm and –70.4 ppm, respectively, and the total experiment time was 13 min.

The gradient type 2D-HOESY experiment performed at 600 MHz (^{1}H reference frequency: 600.13 MHz, ^{19}F reference frequency: 564.69 MHz) was performed using the Bruker default pulse program “hoesygpph” with a mixing time (D8) of 1 s. The number of scans (NS) was set to 32, the sampling point in the t2 domain (F2: TD) and sampling point in the t1 domain (F1: TD) were set to 8192 and 256, the spectra width of ^{19}F (F2: SW) and spectra width of ^{1}H (F1: SW) were set to 20 ppm and 12 ppm, the ^{19}F offset (F2: O1P) and ^{1}H offset (F1: O1P) were set to –70.7 ppm and 4.2 ppm, respectively, and the total experiment time was 11 h.

12. Solvent effect

^{1}H NMR spectrum (400 MHz, CDCl$_3$) of DMTFA

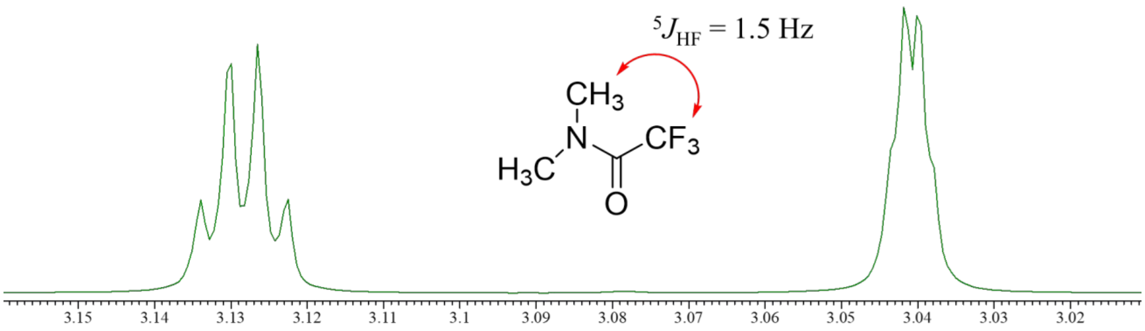

^{1}H NMR spectrum (400 MHz, CD$_3$OD) of DMTFA

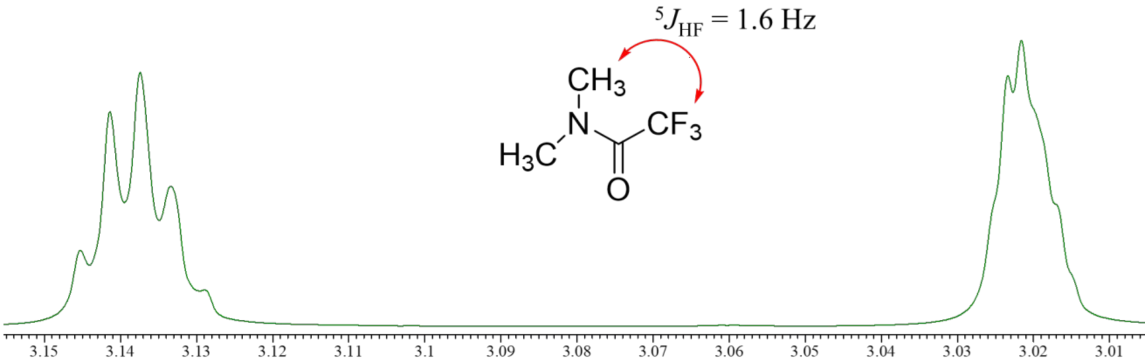

Supplement: Supplementary file 1 — jo3c00311_si_001.pdf [file jo3c00311_si_001.pdf]
